# Trim72 is a major host factor protecting against lethal *Candida albicans* infection

**DOI:** 10.1371/journal.ppat.1012747

**Published:** 2024-11-25

**Authors:** Wang Tan, Jiayu Liu, Renlin Yu, Ping Zhao, Yuhan Liu, Qian Lu, Ke Wang, Hao Ding, Yi Liu, Xiaofei Lai, Ju Cao

**Affiliations:** 1 Department of Laboratory Medicine, The First Affiliated Hospital of Chongqing Medical University, Chongqing, China; 2 Department of Laboratory Medicine, The Seventh People’s Hospital of Chongqing, Central Hospital Affiliated to Chongqing University of Technology, Chongqing, China; 3 Department of Surgery, School of Medicine, Stanford University, Stanford, California, United States of America; University of California Los Angeles David Geffen School of Medicine, UNITED STATES OF AMERICA

## Abstract

*Candida albicans* is the most common aetiologic pathogen of fungal infections associated with high mortality in immunocompromised patients. There is an urgent need to develop new antifungal therapies owing to the poor efficacy and resistance of current antifungals. Here, we report that Trim72 positively regulates antifungal immunity during lethal fungal infection. Trim72 levels are significantly increased after *Candida albicans* infection. In vivo, Trim72 knockout significantly increases mortality, organ fungal burden and kidney damage in mice after lethal *Candida albicans* infection. Whereas recombinant Trim72 protein treatment protects mice against invasive candidiasis. Mechanistically, Trim72 facilitates macrophage infiltration and CCL2 production, which mediates Trim72-elicited protection against lethal *Candida albicans* infection. Furthermore, Trim72 may enhance macrophage migration and CCL2 production via NF-κB and ERK1/2 signaling. Inhibition of NF-κB and ERK1/2 signaling abrogates Trim72-mediated protection against lethal *Candida albicans* infection. Therefore, these data imply that Trim72 may be developed as a host-directed therapy for treating severe systemic candidiasis.

## Author summary

*Candida albicans* is an important cause of fungal infections associated with high mortality rates, and the lack of effective treatment options emphasizes the urgency to develop new antifungal therapies. Trim72 has potential therapeutic effect in a variety of human diseases, however the role of Trim72 in fungal infections is completely unknown. Here we demonstrate that Trim72 may protect against fungal infection by enhancing macrophage recruitment through NF-κB and ERK1/2 signaling-mediated increased cell migration and CCL2 generation in macrophages. Our study not only provides new insights into the regulation of host innate antifungal defense, but also a novel conceptual framework for the development of novel antifungal approaches.

## Introduction

Fungal infections are increasingly recognized as a major public health threat, particularly in immunocompromised patients, such as transplant recipients and those with primary immunodeficiencies and malignancies [1–5]. *Candida* species are the second prevalent agents of fungal infections worldwide. Of these, *Candida albicans* (*C*. *albicans*) is the most common opportunistic aetiologic pathogen causing bloodstream infections and fungal sepsis [6], which can invade and damage solid organs. There are approximately 400,000 cases of bloodstream infections caused by *Candida* species worldwide each year, with mortality exceeding 40% despite the use of antifungal drugs [7,8]. Considering the limited effectiveness of existing therapies and increasing pathogen resistance to antifungal agents [9], gaining mechanistic insights into the immune response against fungi is important for the discovery of new antifungal therapies.

During fungal infections, macrophages are at the first line of defense against *C*. *albicans* and are important for activating and regulating the innate immune response [10]. Following *C*. *albicans* invasion, monocytes/macrophages can be rapidly recruited to the infected site via chemokines, such as CCL2, to clear pathogens though various antifungal responses [11,12]. After recognizing the invading fungus, macrophages triggered the activation of NF-κB and MAPK pathways, leading to the production of cytokines and other mediators to promote fungal clearance [13–15]. Thus, when monocyte/macrophage responses are triggered upon fungal infections, they are recruited to the infected sites and release cytokines and chemokines to attract more immune cells. Based on these factors, macrophages recruitment and chemokine release are the important elements in promoting antifungal immunity.

Tripartite motif protein 72 (Trim72), also known as MG53, is expressed dominantly in the heart and skeletal muscle, participating in multiple physiologic and pathologic processes [16]. Trim72 has been recognized as a potential therapeutic target in protecting a variety of oxygen-dependent organs [17–23]. Besides, Trim72 administration could protect the heart against sepsis-induced myocardial dysfunction by upregulating PPAR*α* expression [24]. Trim72 knockout increased morbidity accompanied by accumulated CD45^+^ cells and elevation of IFN-β in the lung of mice after influenza virus infection [25]. In a murine pneumonia model, Trim72 knockout increased pathogen clearance, reduced cytokine storm, and improved survival via promoting phagocytosis in alveolar macrophages [26]. However, the role of Trim72 in the pathogenesis of fungal infections is completely unknown.

Here we reported that Trim72 protected mice from lethal systemic *C*. *albicans* infection, and that this protection appeared to be due to enhanced macrophage recruitment, partially via increasing CCL2 production in macrophages. Depleting macrophages or blocking CCL2 production abolished the protective effect of Trim72 on lethal against *C*. *albicans* infection. In vitro, Trim72 boosted migration capacity and CCL2 production via NF-κB and ERK1/2 signaling pathways in macrophages. Moreover, inhibition of these signaling eliminated Trim72-medaited protection against *C*. *albicans* infection. This study may provide a theoretical basis for the treatment of fungal infections by targeting Trim72.

## Results

### Trim72 expression is upregulated during *C*. *albicans* infection

**Fig 1:**
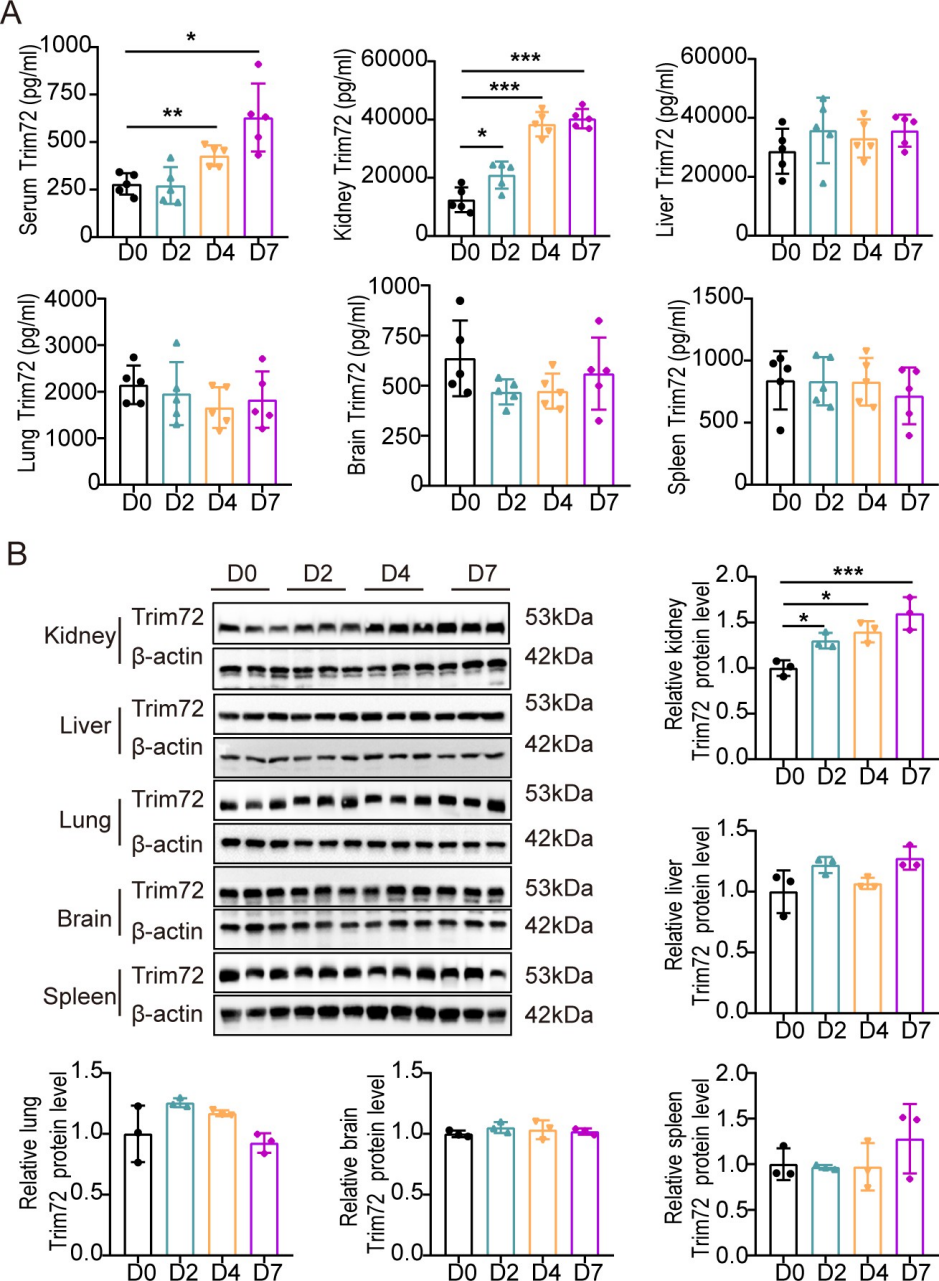
Trim72 expression is upregulated during *Candida albicans* (*C*. *albicans*) infection. (A) Trim72 levels were measured by ELISA in mice serum and tissue homogenates at the indicated times after *C*. *albicans* infection (n = 5 per group). (B) Western blot analysis of Trim72 protein expression in tissue homogenates at the indicated times after *C*. *albicans* infection (n = 3 per group). The intensity of the proteins was measured. Data are representative of triplicate independent experiments. Statistical significance was calculated by kruskal-wallis test or one-way ANOVA followed by Dunnett’s or Dunnett T3 multiple comparison test (A, B). Data are presented as mean ± SD. *p < 0.05, **p < 0.01, ***p < 0.001.

To investigate the expression of Trim72 during *C*. *albicans* infection, we established a systemic candidiasis model by intravenous injection of *C*. *albicans* [27]. Compared to naïve mice, Trim72 concentrations were significantly increased in serum at 4 and 7 days and in kidney tissue homogenates from 2 to 7 days after systemic *C*. *albicans* infection (Fig 1A and 1B), whereas Trim72 levels in the lungs, livers, spleens and brains did not change significantly upon *C*. *albicans* infection (Fig 1A and 1B). Together, these results indicate that Trim72 expression levels are upregulated during *C*. *albicans* infection, implying that Trim72 might play a potential role in the antifungal process.

### *Trim72*^*-/-*^ mice are susceptible to systemic *C*. *albicans* infection

**Fig 2:**
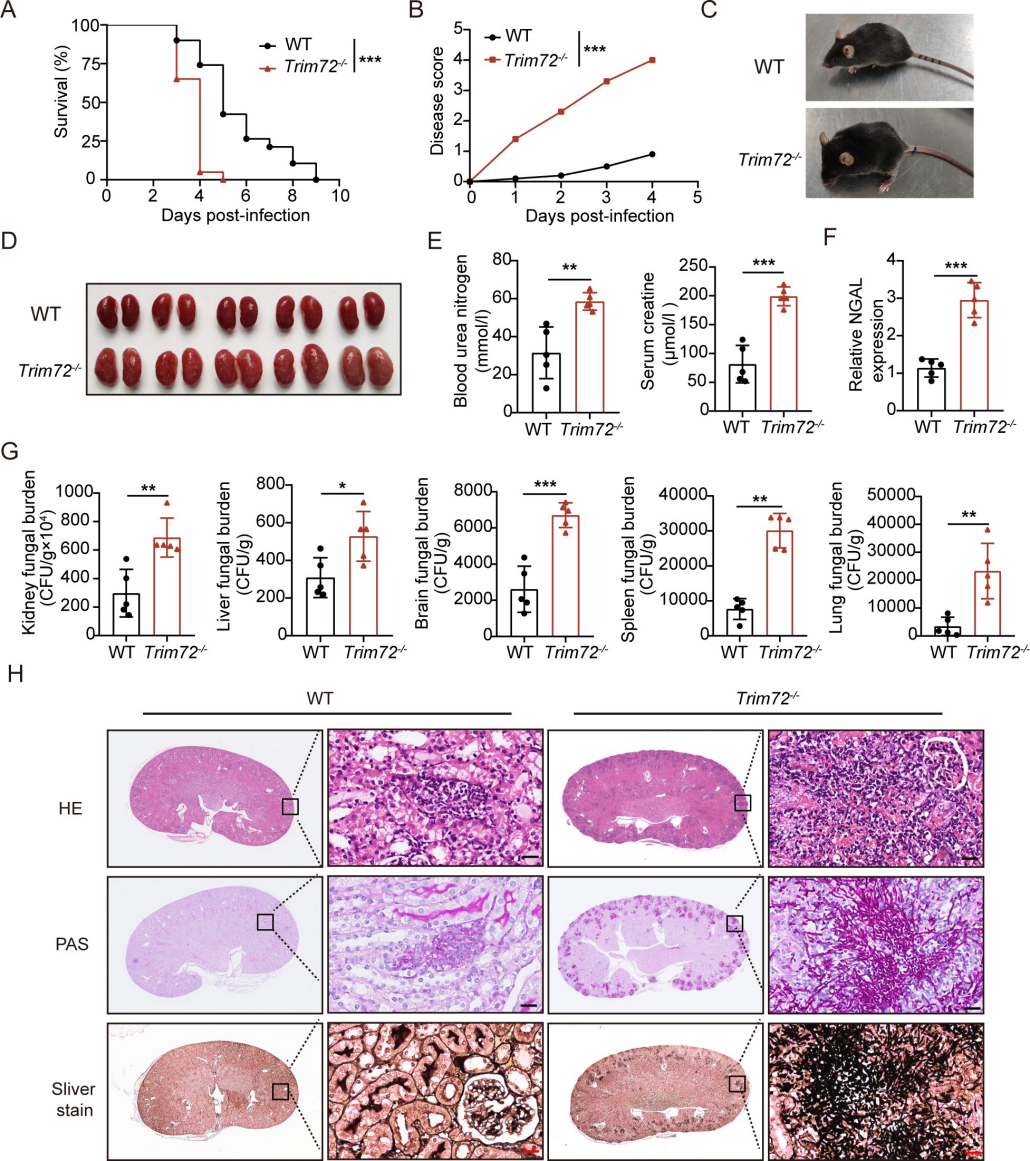
*Trim72*^-/-^ mice were susceptible to systemic *C*. *albicans* infection. (A) WT or *Trim72*^*-/-*^ mice were injected intravenously with 3×10^5^ CFU of *C*. *albicans*. Survival was recorded (n = 20 per group). (B) The disease score of WT and *Trim72*^*-/-*^ mice was recorded at the indicated times after infection (n = 10 per group). (C) Typical pictures of the condition of WT and *Trim72*^*-/-*^ mice were shown at 2 days after infection. (D) Gross picture of the kidney from WT and *Trim72*^*-/-*^ mice at 2 days after infection (n = 5 per group). (E) Blood urea nitrogen and serum creatinine levels in WT and *Trim72*^*-/-*^ mice at 2 days after infection (n = 5 per group). (F) NGAL mRNA expression in kidney tissue from WT and *Trim72*^*-/-*^ mice at 2 days after infection (n = 5 per group). (G) *C*. *albicans* fungal load in organs at 2 days after infection (n = 5 per group). (H) Kidney sections were stained with hematoxylin and eosin (H&E), periodic-acid-Schiff (PAS), or sliver stain at 2 days after *C*. *albicans* infection. Representative pictures were presented. Scale bar = 20 um. Data are representative of triplicate independent experiments. Statistical significance was calculated by Log-rank test (A), two-way ANOVA (B), two-tailed unpaired t-test (E-G) or nonparametric Mann Whitney U test (G). Data are presented as mean (B) or mean ± SD. *p < 0.05, **p < 0.01, ***p < 0.001.

To characterize the potential role of Trim72 against systemic *C*. *albicans* infection in vivo, we challenged wile type (WT) and *Trim72* knockout (*Trim72*^*-/-*^) mice with *C*. *albicans* systemically. *Trim72*^*-/-*^ mice were generated using the clustered regularly interspaced short palindromic repeats (CRISPR) method (S1A Fig). Loss of Trim72 in *Trim72*^*-/-*^ mice was verified by PCR and protein blot analysis (S1B and S1C Fig). *Trim72*^*-/-*^ mice were more susceptible to systemic *C*. *albicans* infection than WT controls, as indicated by worse survival (Fig 2A), higher disease scores (Fig 2B), and poorer physical condition (Fig 2C).

Given that the kidney is a primary site of *C*. *albicans* infiltration in mice leading to abscess formation and renal failure [28], the increased mortality in *Trim72*^*-/-*^ mice was probably due to the increased evidence of kidney damage and decreased clearance of *C*. *albicans* from the kidney. As expected, the kidneys of *Trim72*^*-/-*^ mice were more enlarged, swollen, pale and had more prominent nodules (Fig 2D). In accordance with the general appearance of the kidneys, *Trim72*^*-/*-^ mice had elevated serum concentrations of blood urea nitrogen and serum creatinine concentrations (Fig 2E), and upregulated neutrophil gelatinase-associated liposomes (NGAL) in renal homogenates (Fig 2F) at 2 days after *C*. *albicans* infection compared to WT controls. Among these, NGAL is a marker of tubular epithelial damage for the early diagnosis of acute kidney injury (AKI) [29]. Accordingly, *Trim72*^*-/-*^ mice had higher fungal burdens in the kidneys and other substantive organs (liver, brain, spleen, lung) than WT control mice (Fig 2G). Histopathologic analysis also demonstrated that the kidneys of *Trim72*^*-/-*^ mice exhibited increased renal inflammation and more *C*. *albicans* burden at 2 days after infection (Fig 2H). Taken together, these results suggested that Trim72 deficiency may impair host immune response against *C*. *albicans* infection in mice.

### Trim72 treatment protects mice against lethal *C*. *albicans* infection

**Fig 3:**
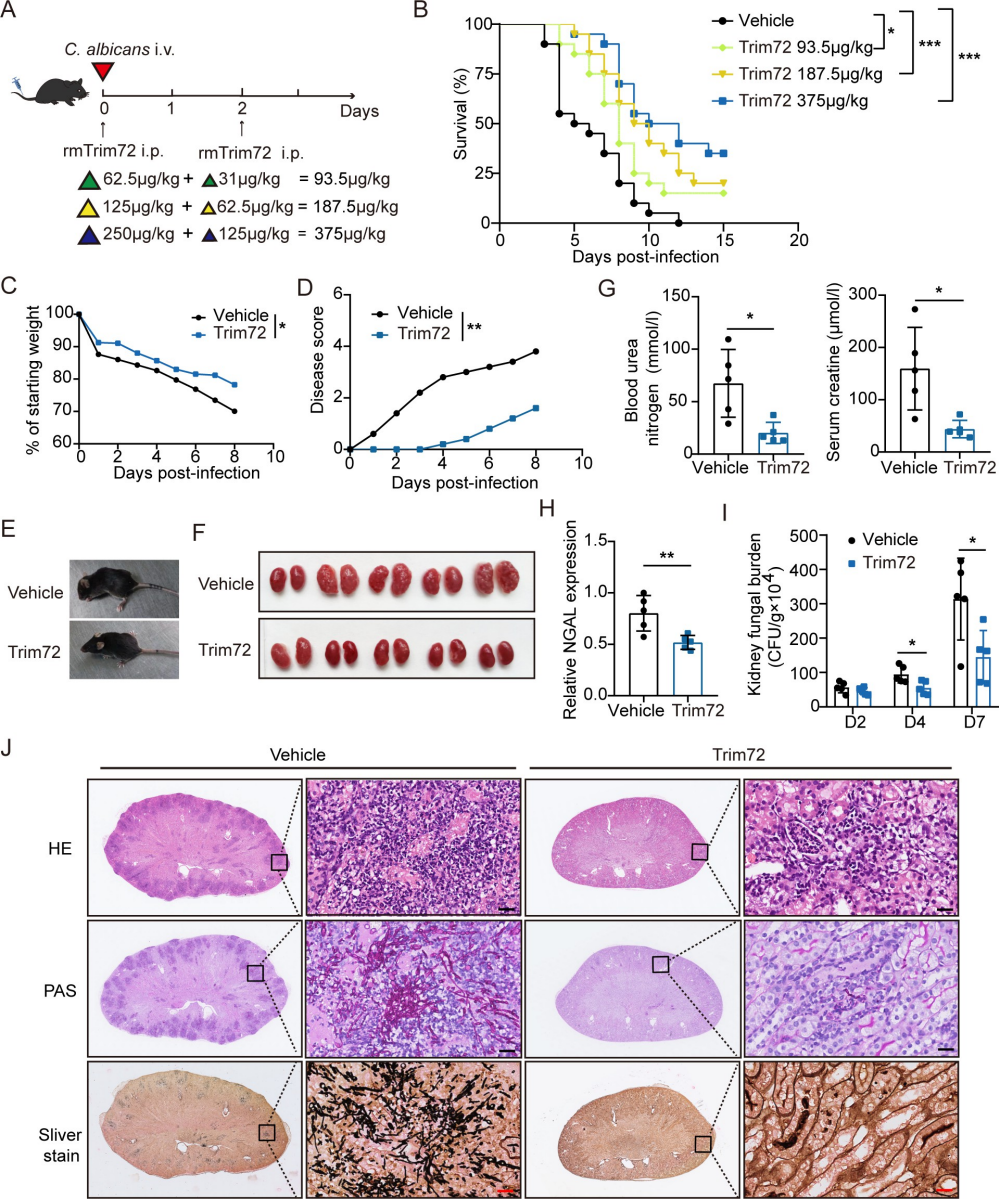
Trim72 treatment protects mice against *C*. *albicans* infection. (A) The study protocol was shown. Mice were injected intraperitoneally with various doses of recombinant murine Trim72 (rmTrim72) (62.5, 125, and 250 μg/kg) or vehicle control 30 min prior to intravenous infection with 3×10^5^ CFU *C*. *albicans*, and with 31, 62.5, and 125 μg/kg rmTrim72 respectively at 2 days after infection. (B) Survival of vehicle-treated and various doses of rmTrim72-treated mice (n = 20 per group). (C and D) Weight loss (n = 10 per group) (C) and disease score (n = 5 per group) (D) of vehicle-treated and rmTrim72 (375μg/kg)-treated mice at the indicated times after infection. (E) Typical pictures of the condition of vehicle-treated and rmTrim72 (375μg/kg)-treated mice at 7 days after infection. (F) Gross picture of the kidney from vehicle-treated and rmTrim72 (375μg/kg)-treated mice at 7 days after infection (n = 5 per group). (G) Blood urea nitrogen and serum creatinine levels in vehicle-treated and rmTrim72 (375μg/kg)-treated mice at 7 days after infection (n = 5 per group). (H) NGAL mRNA expression in kidney tissue from vehicle-treated and rmTrim72 (375μg/kg)-treated mice at 7 days after infection (n = 5 per group). (I) *C*. *albicans* fungal load in kidney from vehicle-treated and rmTrim72 (375μg/kg)-treated mice at indicated times after infection (n = 5 per group). (J) Kidney sections were stained with H&E, PAS or sliver stain at 7 days after infection. Representative images were shown. Scale bar = 20 um. All data are representative of triplicate independent experiments. Statistical significance was calculated by Log-rank test (B), two-way ANOVA (C and D), two-tailed unpaired t-test (G-I). Data are presented as mean (C and D) or mean ± SD. *p < 0.05, **p < 0.01, ***p < 0.001.

To further investigate whether Trim72 replenishment could modify the progression of *C*. *albicans* infection, we treated WT mice with different doses of recombinant murine (rm) Trim72 protein (62.5, 125 or 250 μg/kg) upon systemic *C*. *albicans* infection, and injected 31, 62.5, 125 μg/kg rmTrim72 respectively at 2 days after infection (Fig 3A). Treatment with 375 μg/kg (250 μg/kg + 125 μg/kg) of rmTrim72 protein maximized survival in mice compared to other therapeutic doses (Fig 3B). Given that, our subsequent experiments were carried out with 375 μg/kg rmTrim72. Consistent with survival, Trim72 treatment resulted in significantly less body weight loss (Fig 3C), lower disease scores (Fig 3D), and better physical condition (Fig 3E).

Next, we investigated whether Trim72 supplementation could reduce renal injury and renal fungal load in mice after systemic *C*. *albicans* infection. Unlike the kidneys from vehicle-treated controls, kidneys from the Trim72-treated mice were smaller, less grossly pathologically swollen and pale, and had less prominent nodules at 7 days after infection (Fig 3F). In addition, serum concentrations of blood urea nitrogen and serum creatinine, as well as NGAL expression levels in renal tissue, were significantly lower in Trim72-treated mice than in vehicle-treated mice at 7 days after infection (Fig 3G and 3H). Furthermore, the kidney fungal loads in Trim72-treated mice were lower at 2 days after infection, which reached statistical significance at 4 and 7 days compared to vehicle-treat mice (Fig 3I). The fungal load in other organs such as liver, lung and spleen in Trim72-treated mice also showed a decreasing tendency at 2 days, declined significantly at 4 days, and was basically cleared at 7 days post-infection (S2 Fig). Histopathological analysis also demonstrated that Trim72 treatment decreased renal inflammation and fungal burden at 7 days after infection (Fig 3J). These data confirm that Trim72 might be a potential therapeutic target for lethal *C*. *albicans* infection.

### Trim72 promotes monocyte/macrophage recruitment and CCL2 production in the infected kidney

**Fig 4:**
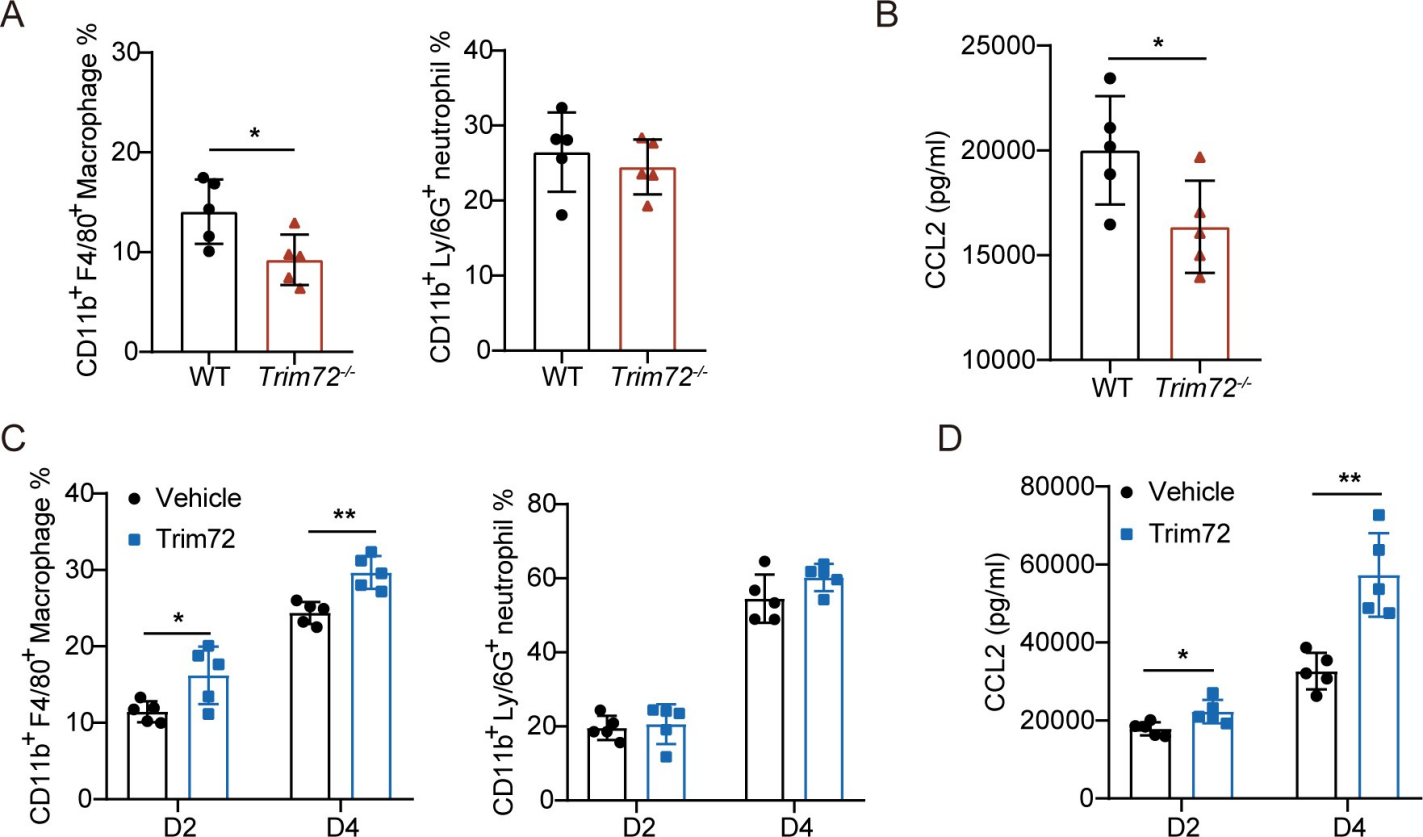
Trim72 promotes macrophage recruitment and CCL2 production in the infected kidney. (A) Flow cytometry analysis of CD11b^+^F4/80^+^ macrophages and CD11b^+^Ly6G^+^ neutrophils in the kidneys of WT or *Trim72*^*-/-*^ mice at 2 days after *C*. *albicans* infection (n = 5 per group). (B) CCL2 levels in renal tissue homogenates from WT or *Trim72*^*-/-*^ mice detected by ELISA at 2 days after *C*. *albicans* infection (n = 5 per group). (C) Flow cytometry analysis of CD11b^+^F4/80^+^ macrophages and CD11b^+^Ly6G^+^ neutrophils in the kidneys of vehicle-treated or rmTrim72 (375μg/kg)-treated mice at 2 and 4 days after *C*. *albicans* infection (n = 5 per group). (D) CCL2 levels in renal tissue homogenates from vehicle-treated or rmTrim72 (375μg/kg)-treated mice detected by ELISA at 2 and 4 days after *C*. *albicans* infection (n = 5 per group). Data are representative of triplicate independent experiments. Statistical significance was calculated by two-tailed unpaired t-test (A-D). Data are presented as mean ± SD. *p < 0.05, **p < 0.01.

In mice, after infection with *C*. *albicans*, *C*. *albicans* can cross the blood vessel walls and invade the kidney, triggering the recruitment of immune cells, particularly monocytes/macrophages and neutrophils, which are capable of clearing the fungus from the kidneys [30]. We first tested whether Trim72 deficiency affects innate immune cell development by monitoring the immune cell abundance in the spleen and bone marrow of unchallenged WT and *Trim72*^*-/-*^ mice. We found that the total number or cell composition of immune cells in these organs was similar between WT and *Trim72*^*-/-*^ mice (S3A and S3B Fig), suggesting Trim72 is dispensable for innate immune development. We next evaluated the effect of Trim72 on monocyte/macrophage and neutrophil infiltration in the murine *C*. *albicans* infection model. Trim72 deficiency impeded the recruitment of CD11b^+^F4/80^+^ macrophages into the kidneys at 2 days after *C*. *albicans* infection compared to WT mice (Figs 4A and S4A). Immunofluorescence provided a more visual indication of reduced macrophage infiltration in the infected kidney of *Trim72*^*-/-*^ mice relative to WT mice after *C*. *albicans* infection (S4B Fig). However, no significant differences in CD11b^+^Ly6G^+^ neutrophil accumulation were observed between the kidneys of WT and *Trim72*^*-/-*^ mice after *C*. *albicans* infection (Figs 4A and S4A).

CCL2 acts as a major chemokine implicated in attracting monocytes/ macrophages to infected sites [31]. Having demonstrated that Trim72 deficiency negatively regulates macrophage recruitment to the infected kidney, we next studied whether Trim72 deficiency affects CCL2 production. At 2 days after *C*. *albicans* infection, Trim72 deficiency decreased CCL2 concentration compared to the WT group (Fig 4B), while other cytokines and chemokines, including IL-1β, IL-6, TNF-α, IL-4 and CXCL1 were not significantly changed (S4C Fig), indicating that Trim72 may have a selective effect on CCL2 production, which may modulate the pathological process of *C*. *albicans* infection.

To rule out the possibility that the changes in monocyte/macrophage recruitment and cytokine expression were caused by differences in fungal load, we analyzed macrophage recruitment and cytokine expression levels when kidney fungal abundance was similar (at 2 days) or not similar (at 4 days) in Trim72-treated mice. In agreement with the above findings, Trim72 treatment significantly enhanced macrophage recruitment in the infected kidney (Figs 4C, S5A and S5B), and selectively increased CCL2 production at 2 and 4 days after *C*. *albicans* infection (Fig 4D), while did not affect neutrophil accumulation and TNF-α, IL-1β, IL-6, CXCL1, and IL-4 production in the infected kidney (Figs 4C, S5A and S5C). Together, these results suggest that Trim72 promotes macrophage recruitment and CCL2 production in the infected kidney upon *C*. *albicans* infection.

### CCL2–macrophage axis mediates Trim72-elicitated protection against systemic *C*. *albicans* infection in mice

**Fig 5:**
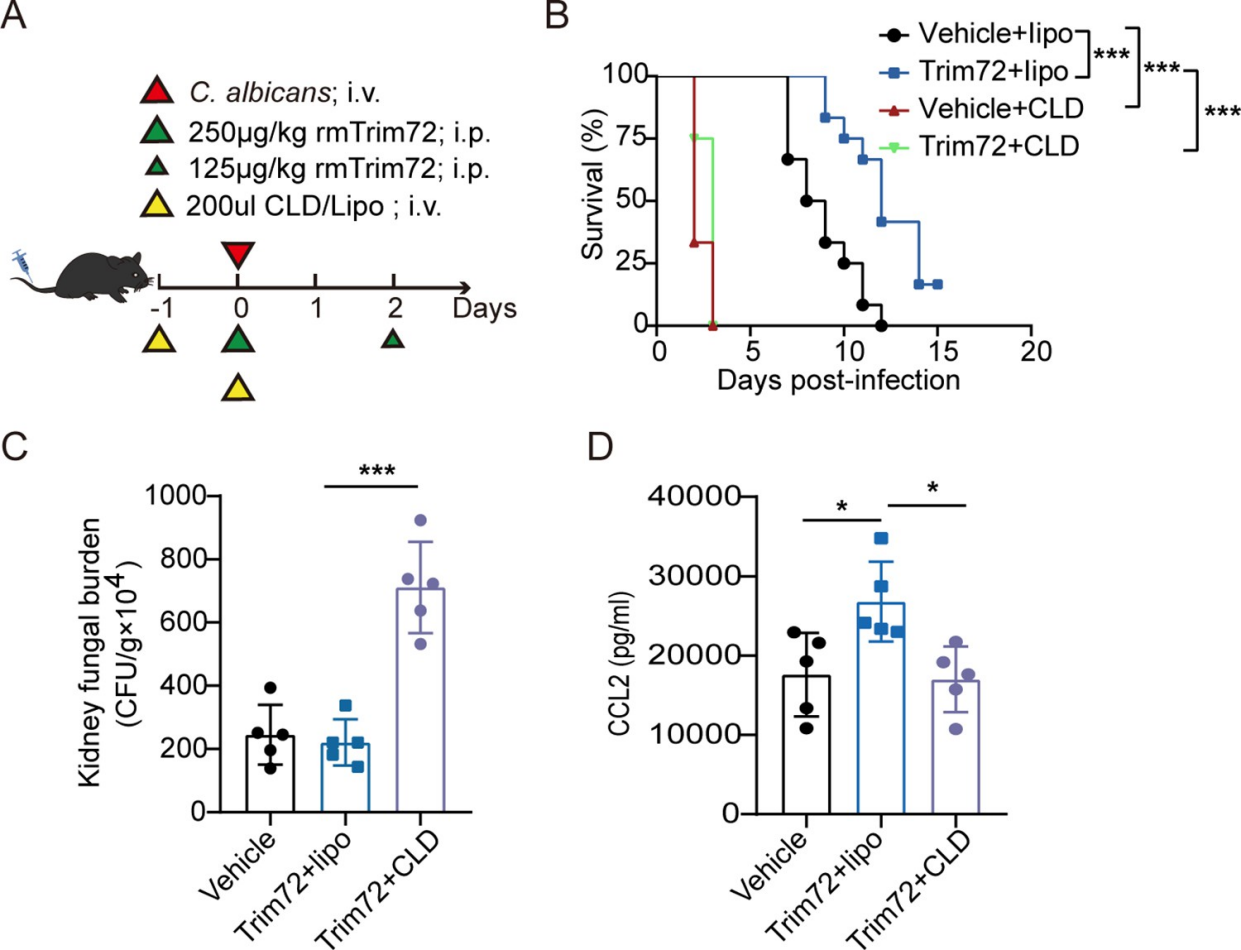
Macrophage-mediated Trim72 provides protection against systemic *C*. *albicans* infection in mice. (A) The study protocol for the administration of rmTrim72 (375μg/kg) and 200ul of clodronate-containing liposomes (CLD) or empty liposomes (Lipo) was shown. Mice were intravenous infected with 3×10^5^ CFU of *C*. *albicans*. (B) Survival of vehicle-treated or rmTrim72 (375μg/kg)-treated mice in the presence or absence of macrophages depletion after *C*. *albicans* infection (n = 12 per group). (C and D) *C*. *albicans* fungal load (C) and CCL2 levels (D) in the kidneys from mice in (A) at 2 days after infection (n = 5 per group). Survival data were collected from three independent experiments. Other data are representative of triplicate independent experiments. Statistical significance was calculated by Log-rank test (B), or one-way ANOVA followed by Dunnett’s multiple comparison test (C, D). Data are presented as mean ± SD. *p < 0.05, **p < 0.01, ***p < 0.001.

To investigate whether macrophages are responsible for Trim72-mediated protection against *C*. *albicans* infection, we depleted macrophages with clodronate liposomes in Trim72-treated mice (Figs 5A and S6). Flow cytometry analysis confirmed dramatically reduced numbers of CD11b^+^F4/80^+^ macrophages in kidney tissues after macrophage depletion (S6 Fig). Depleting macrophages abolished the beneficial effects of Trim72 on survival and kidney fungal clearance in *C*. *albicans*-infected mice (Fig 5B and 5C). In addition, macrophage depletion abrogated Trim72-enhanced CCL2 production in kidney tissues (Fig 5D), suggesting that macrophages might be a major source of CCL2 elicited by Trim72 in systemic *C*. *albicans* infection.

**Fig 6:**
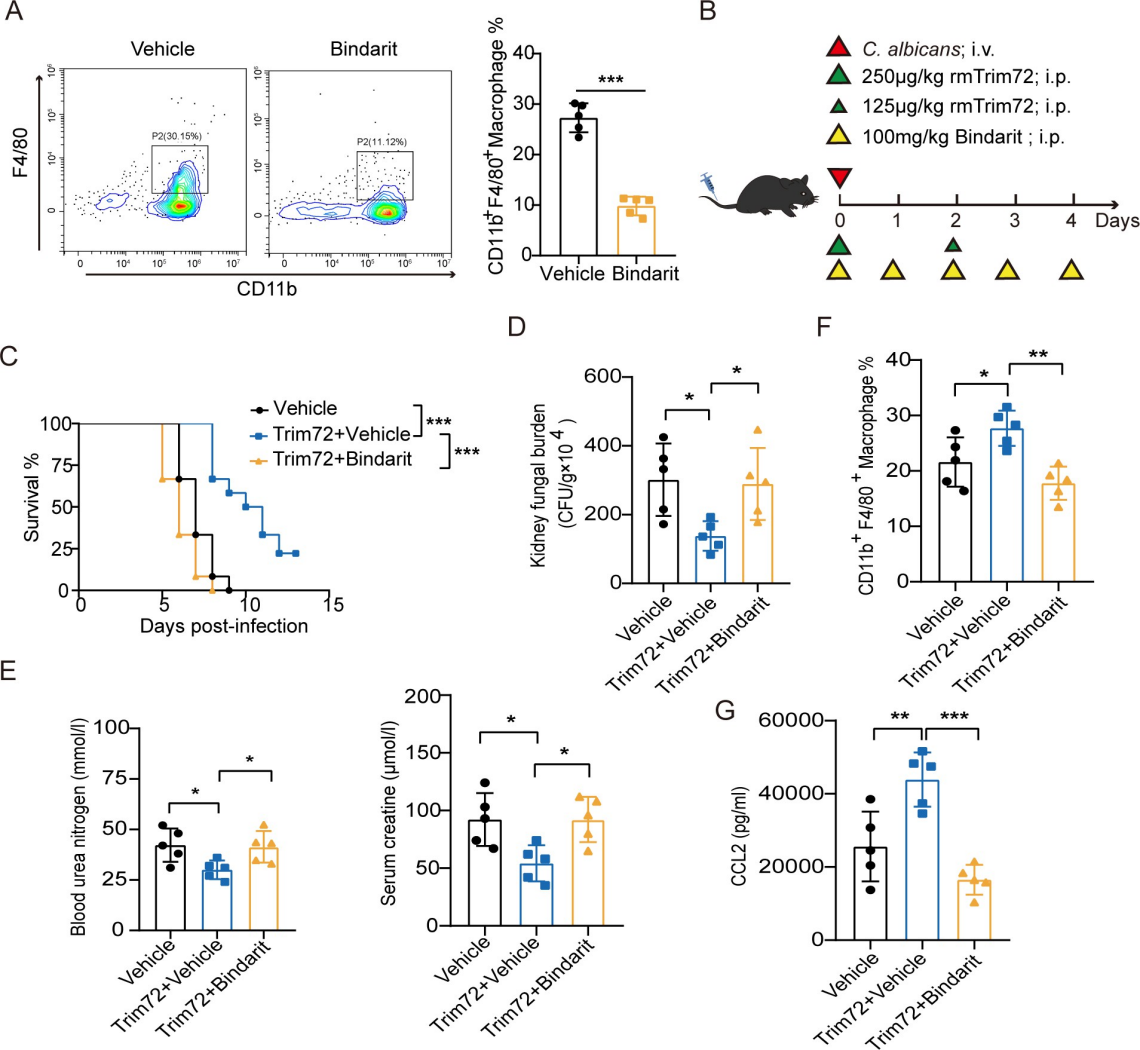
CCL2–macrophage axis mediates Trim72-elicitated protection against systemic *C*. *albicans* infection in mice. (A) Flow cytometry analysis of CD11b^+^F4/80^+^ macrophages in the kidneys of vehicle-treated or Bindarit (100mg/kg)-treated mice at 4 days after *C*. *albicans* infection (n = 5 per group). (B) The study protocol for the administration of rmTrim72 (375μg/kg) and Bindarit (100mg/kg) was shown. (C) Survival of mice in (B) (n = 12 per group). (D) *C*. *albicans* fungal load in the kidneys from mice in (B) at 4 days after infection (n = 5 per group). (E) Blood urea nitrogen and serum creatinine concentrations in mice in (B) at 4 days after infection (n = 5 per group). (F) CD11b^+^F4/80^+^ macrophages in the kidneys of mice in (B) at 4 days after infection by flow cytometry analysis (n = 5 per group). (G) CCL2 levels in renal tissue homogenates of mice in (B) detected by ELISA at 4 days after infection (n = 5 per group). Survival data were collected from three independent experiments. Other data are representative of triplicate independent experiments. Statistical significance was calculated by two-tailed unpaired t-test (A), Log-rank test (C), or one-way ANOVA followed by Dunnett’s multiple comparison test (D-G). Data are presented as mean ± SD. *p < 0.05, **p < 0.01, ***p < 0.001.

To further investigate the contribution of CCL2-macrophage axis to Trim72-mediated protection against lethal *C*. *albicans* infection, we used the CCL2 inhibitor, Bindarit, to block the process of CCL2 [32], which reduced the influx of macrophages into the infected kidneys (Fig 6A). Administration of Bindarit significantly reduced survival (Fig 6B and 6C), increased renal fungal load (Fig 6D) and increased blood urea nitrogen and serum creatinine concentrations (Fig 6E) in Trim72-treated mice after *C*. *albicans* infection. In addition, Bindarit administration reduced macrophage recruitment and CCL2 expression in the kidneys of Trim72-treated mice (Fig 6F and 6G), indicating that Trim72 promoted macrophage recruitment, which may be associated with upregulated CCL2 expression. Together, these data suggest that CCL2-macrophage axis is, at least in part, responsible for the protection of mice against systemic *C*. *albicans* infection elicited by Trim72.

### Trim72 enhances macrophage migration and CCL2 production in vitro

**Fig 7:**
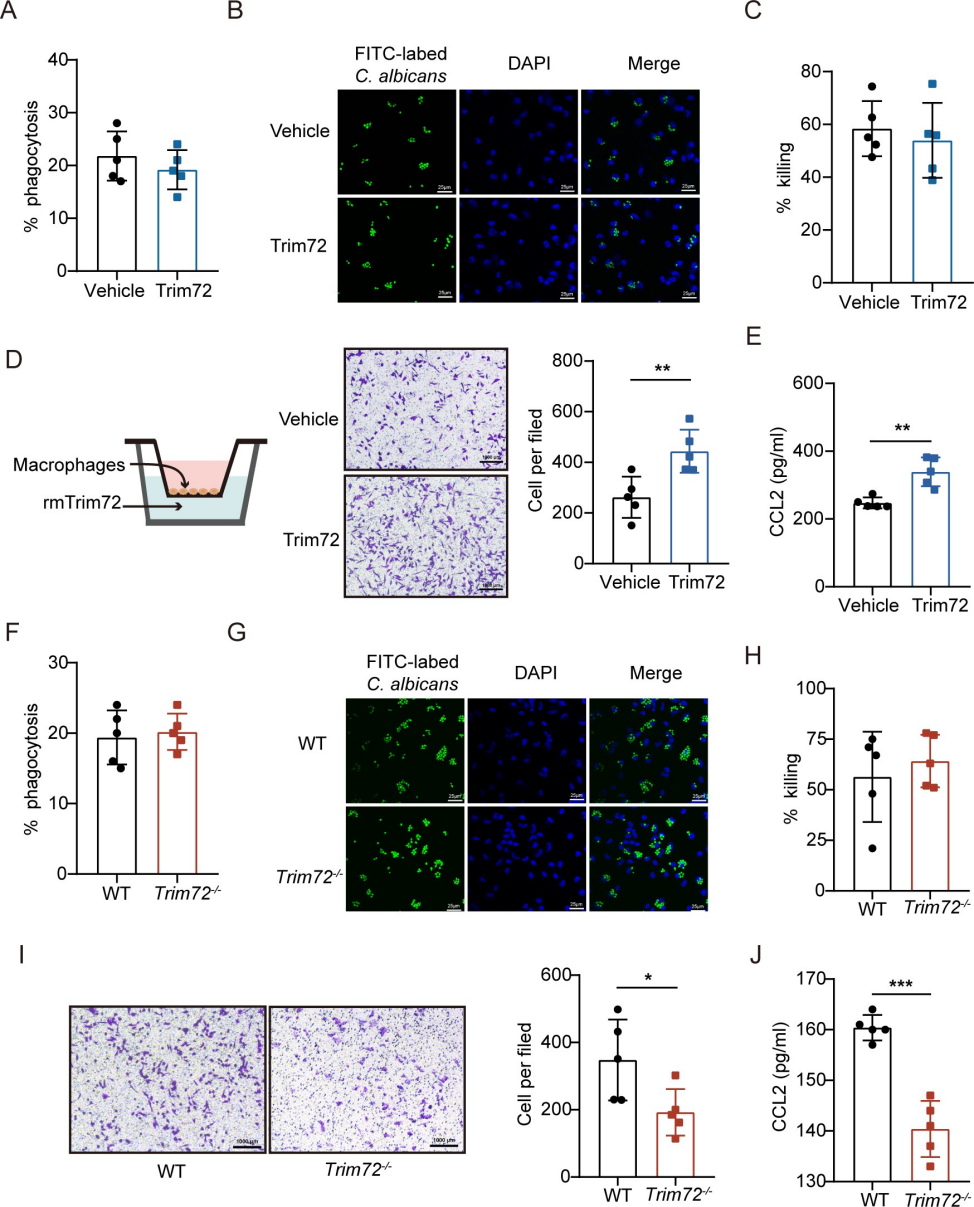
Trim72 enhances macrophage migration and CCL2 production in vitro. (A) Rates of phagocytosis for live *C*. *albicans* (MOI = 1) by peritoneal macrophages pretreated with vehicle or rmTrim72 (1μg/ml) overnight (n = 5 per group). (B) Phagocytosis for FITC-labeled heat-killed *C*. *albicans* (MOI = 1) by peritoneal macrophages pretreated with vehicle or rmTrim72 (1μg/ml) overnight (n = 5 per group). Representative images were shown. Scale bar = 25 um. (C) Rates of killing for live *C*. *albicans* (MOI = 1) by peritoneal macrophages pretreated with vehicle or rmTrim72 (1μg/ml) overnight (n = 5 per group). (D) Schematic diagram of the transwell migration assay was shown. Migration of primary peritoneal macrophages treated with vehicle or rmTrim72 (1μg/ml) in a transwell migration assay (n = 5 per group). Representative images were presented. Scale bar = 1000 um. (E) CCL2 levels detected by ELISA in primary peritoneal macrophages treated with vehicle or rmTrim72 (1μg/ml) (n = 5 per group). (F) Rates of phagocytosis for live *C*. *albicans* (MOI = 1) by WT or *Trim72*^*-/-*^ peritoneal macrophages (n = 5 per group). (G) Phagocytosis for FITC-labeled heat-killed *C*. *albicans* (MOI = 1) by WT or *Trim72*^*-/-*^ peritoneal macrophages. Representative images were shown. Scale bar = 25 um. (H) Rates of killing for live *C*. *albicans* (MOI = 1) by WT or *Trim72*^*-/-*^ peritoneal macrophages (n = 5 per group). (I) Migration of primary peritoneal macrophages isolated from WT or *Trim72*^*-/-*^ mice in a transwell migration assay (n = 5 per group). Representative images were shown. Scale bar = 1000 um. (J) CCL2 levels detected by ELISA in primary peritoneal macrophages isolated from WT or *Trim72*^*-/-*^ mice (n = 5 per group). Data are representative of triplicate independent experiments. Statistical significance was calculated by two-tailed unpaired t-test (A-D, F-J) or nonparametric Mann Whitney U test (E). Data are presented as mean ± SD. *p < 0.05, **p < 0.01, ***p < 0.001.

We next explored whether Trim72 regulates fungal clearance by macrophages using recombinant murine Trim72 protein. We found that Trim72 treatment did not affect macrophage phagocytosis of *C*. *albicans* (Fig 7A and 7B) and killing ability (Fig 7C). Having observed that Trim72 increased macrophage influx into the infected kidneys in mice after systemic *C*. *albicans* infection, we next determined whether Trim72 affect macrophage migration in vitro. Trim72 treatment significantly enhanced migration ability of peritoneal macrophages and bone marrow-derived macrophages (BMDM) (Figs 7D, S7A and S7B). Since adhesion receptors play an important role in macrophage recruitment [33], we further analyzed the expression levels of the adhesion receptors from the β2 integrin family: αD, αL, αM, αX and β2 integrin. However, we did not detect any differences in integrin expression between Trim72-treated and vehicle-treated macrophages, implying that Trim72 did not affect adhesion receptors expression (S7C Fig). Next, considering that Trim72 augmented CCL2 production in the infected kidneys and that macrophages may be a major source of CCL2, we further validated the role of Trim72 in CCL2 production in macrophages. As expected, CCL2 production was significantly upregulated in Trim72-treated macrophages compared to vehicle treatment (Fig 7E). Besides, we determined other cytokine levels and found that the levels of IFN-a, IFN-β, IFN-γ were similar between Trim72-treated and vehicle-treated macrophages (S8 Fig).

Consistent with above results, *Trim72*^*-/-*^ macrophages isolated from *Trim72*^*-/-*^ mice showed similar phagocytic uptake (Fig 7F and 7G) and killing capacity (Fig 7H) compared to WT macrophages. In addition, migration capacity and CCL2 production were reduced in *Trim72*^*-/-*^ macrophages (Fig 7I and 7J). Together, these results show that Trim72 promotes macrophage migration and CCL2 production in vitro.

### NF-kB and ERK1/2 signaling pathways regulate macrophage migration and CCL2 production induced by Trim72

**Fig 8:**
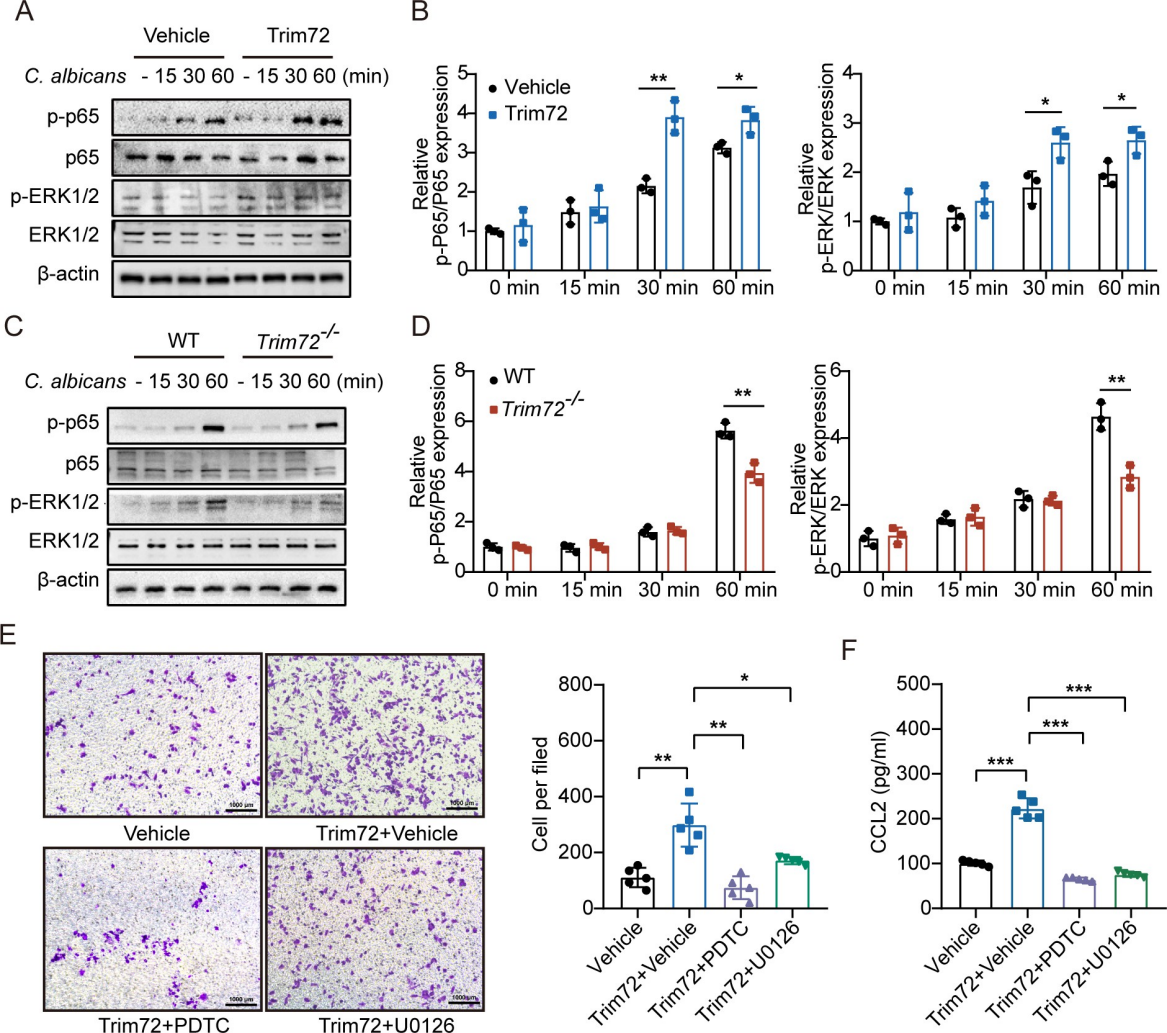
NF-kB and ERK1/2 signaling pathways regulate macrophage migration and CCL2 production induced by Trim72. (A and B) Primary peritoneal macrophages pretreated with rmTrim72 (1μg/ml) or vehicle were stimulated with *C*. *albicans* for the indicated periods and analyzed by Western blotting for the indicated signaling molecules. Representative images were shown (A). The intensity of the proteins was measured (B) (n = 3 per group). (C and D) WT or *Trim72*^*-/-*^ macrophage were stimulated with *C*. *albicans* for the indicated periods and analyzed by Western blotting for the indicated signaling molecules. Representative images were shown (C). The intensity of the proteins was measured (D) (n = 3 per group). (E) Peritoneal macrophages were stimulated with NF-κB inhibitor PDTC (25uM) and ERK1/2 inhibitor U0126 (20uM) or vehicle, followed by rmTrim72 (1μg/ml) or vehicle treatment. Migration of primary peritoneal macrophages was assessed in a transwell migration assay (n = 5 per group). Representative images were shown. Scale bar = 1000μm. (F) CCL2 levels of macrophages in (E) detected by ELISA (n = 5 per group). Data are representative of triplicate independent experiments. Statistical significance was calculated by two-tailed unpaired t-test (B, D), one-way ANOVA followed by Dunnett T3 multiple comparison test (E, F). Data are presented as mean ± SD. *p < 0.05, **p < 0.01, ***p < 0.001.

To gain more insight into the mechanism by which Trim72 regulates CCL2 production and migration capacity of macrophages, we analyzed changes in the signaling pathways that affect antifungal responses including NF-κB and MAPKs (ERK1/2, P38, and JNK) signaling in response to *C*. *albicans* infection in primary mouse peritoneal macrophages. We found that the phosphorylation levels of p65 (Ser536) and ERK1/2 (Thr202/Tyr204) were enhanced in Trim72-treated macrophages as compared to vehicle-treated macrophages in response to *C*. *albicans* infection (Fig 8A and 8B). However, the phosphorylation of JNK (Thr183/Tyr185) and p38 (Thr180/Tyr182) induced by *C*. *albicans* did not differ in the treatment of Trim72 (S9A Fig). Also, Trim72 deficiency attenuated the *C*. *albicans*-induced activation of NF-κB and ERK1/2 signaling pathways (Fig 8C and 8D), and did not affect the phosphorylation of JNK (Thr183/Tyr185) and p38 (Thr180/Tyr182) induced by *C*. *albicans* (S9B Fig).

To further correlate the functional changes modulated by Trim72 treatment with NF-κB and ERK1/2 signaling pathways, we treated macrophages with the inhibitors of NF-κB (PDTC) [34] or ERK1/2 (U0126) [35]. As expected, both NF-κB inhibitor PDTC (25uM) and ERK1/2 inhibitor U0126 (20uM) could impede the increased migration capacity and CCL2 production in macrophages induced by Trim72 (Fig 8E and 8F). Together, these results suggest that, in macrophages, Trim72 enhanced cell migration capacity and CCL2 production modulated by NF-κB and ERK1/2 signaling.

### Inhibiting NF-κB and ERK1/2 signaling pathways abolished Trim72-mediated protection against invasive *C*. *albicans* infection

**Fig 9:**
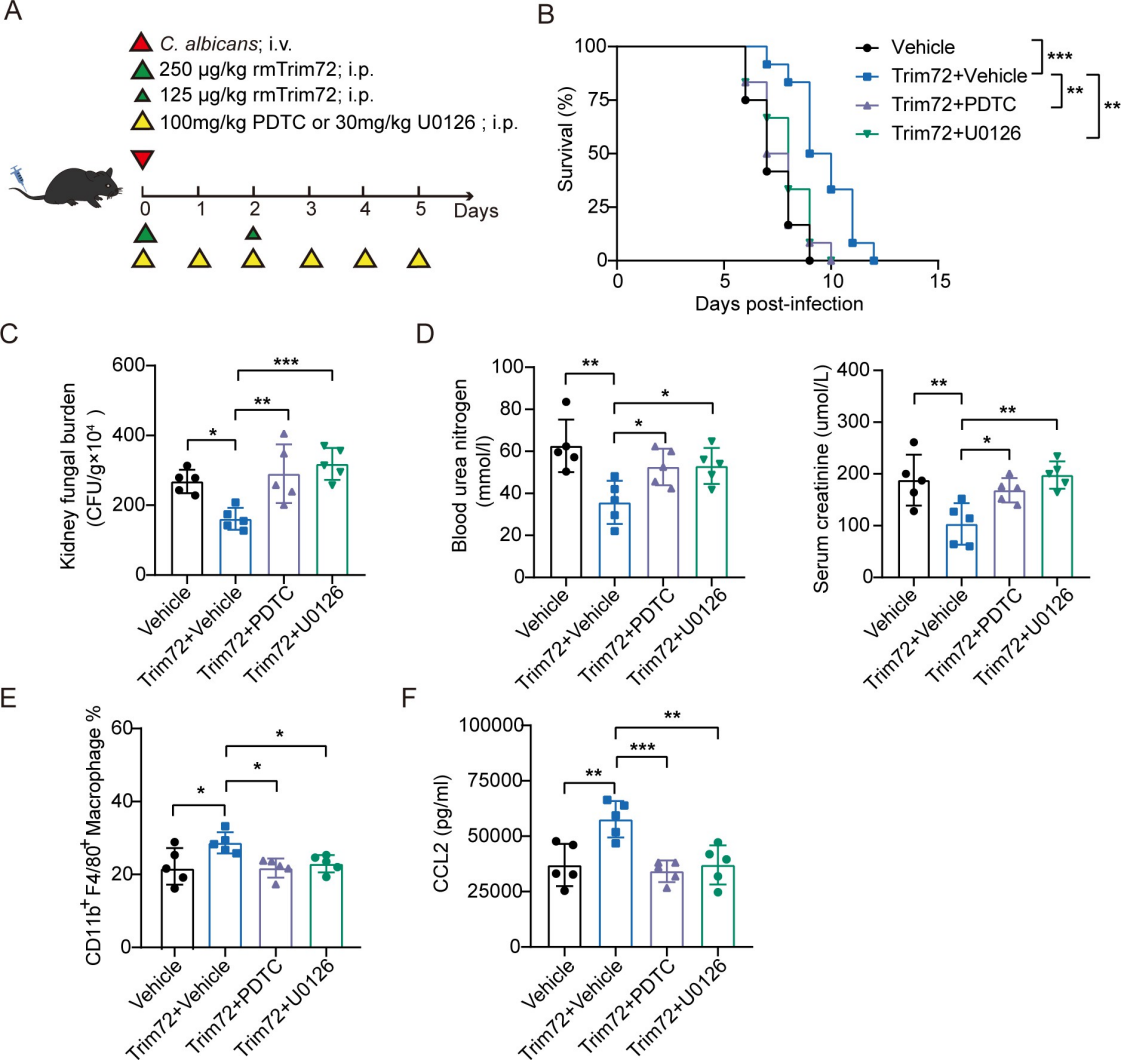
Inhibiting NF-κB and ERK1/2 signaling pathways abolished Trim72-mediated protection against invasive *C*. *albicans* infection. (A) The study protocol for the administration of rmTrim72 (375μg/kg), PDTC (100mg/kg) and U0126 (30mg/kg) was shown. Mice were intravenous infected with 3×10^5^ CFU of *C*. *albicans*. (B) Survival of mice in (A) (n = 12 per group). (C) *C*. *albicans* fungal load in kidneys from mice in (A) at 4 days after infection (n = 5 per group). (D) Blood urea nitrogen and serum creatinine levels in mice in (A) at 4 days after infection (n = 5 per group). (E) CD11b^+^F4/80^+^ macrophages in the kidneys of mice in (A) at 4 days after *C*. *albicans* infection by flow cytometry analysis (n = 5 per group). (F) CCL2 levels in renal tissue homogenates from mice in (A) detected by ELISA at 4 days after *C*. *albicans* infection (n = 5 per group). Survival data were collected from three independent experiments. Other data are representative of triplicate independent experiments. Statistical significance was calculated by Log-rank test (B), or one-way ANOVA followed by Dunnett’s multiple comparison test (C-F). Data are presented as mean ± SD. *p < 0.05, **p < 0.01, ***p < 0.001.

To address the possibility that NF-κB and ERK1/2 signaling pathways may be involved in Trim72-mediated protection against invasive *C*. *albicans* infection, Trim72-treated mice were injected intraperitoneally daily with 100 mg/kg PDTC to inhibit NF-κB signaling or 30 mg/kg U0126 to inhibit ERK1/2 signaling (Fig 9A). Expectedly, administration of PDTC or U0126 significantly worsened the survival of Trim72-treated mice (Fig 9B), with increased kidney fungal burden (Fig 9C) and blood urea nitrogen and serum creatinine levels (Fig 9D). Moreover, the increased levels of macrophage accumulation and CCL2 production in the infected kidneys induced by Trim72 were alleviated by PDTC or U0126 treatment after *C*. *albicans* infection (Fig 9E and 9F). Together, these data further indicate that NF-κB and ERK1/2 signaling pathways mediate Trim72 protection against invasive *C*. *albicans* infection.

### Trim72 augments migration capacity and CCL2 production through NF-κB and ERK1/2 signaling in human monocyte-derived macrophages (hMDMs)

**Fig 10:**
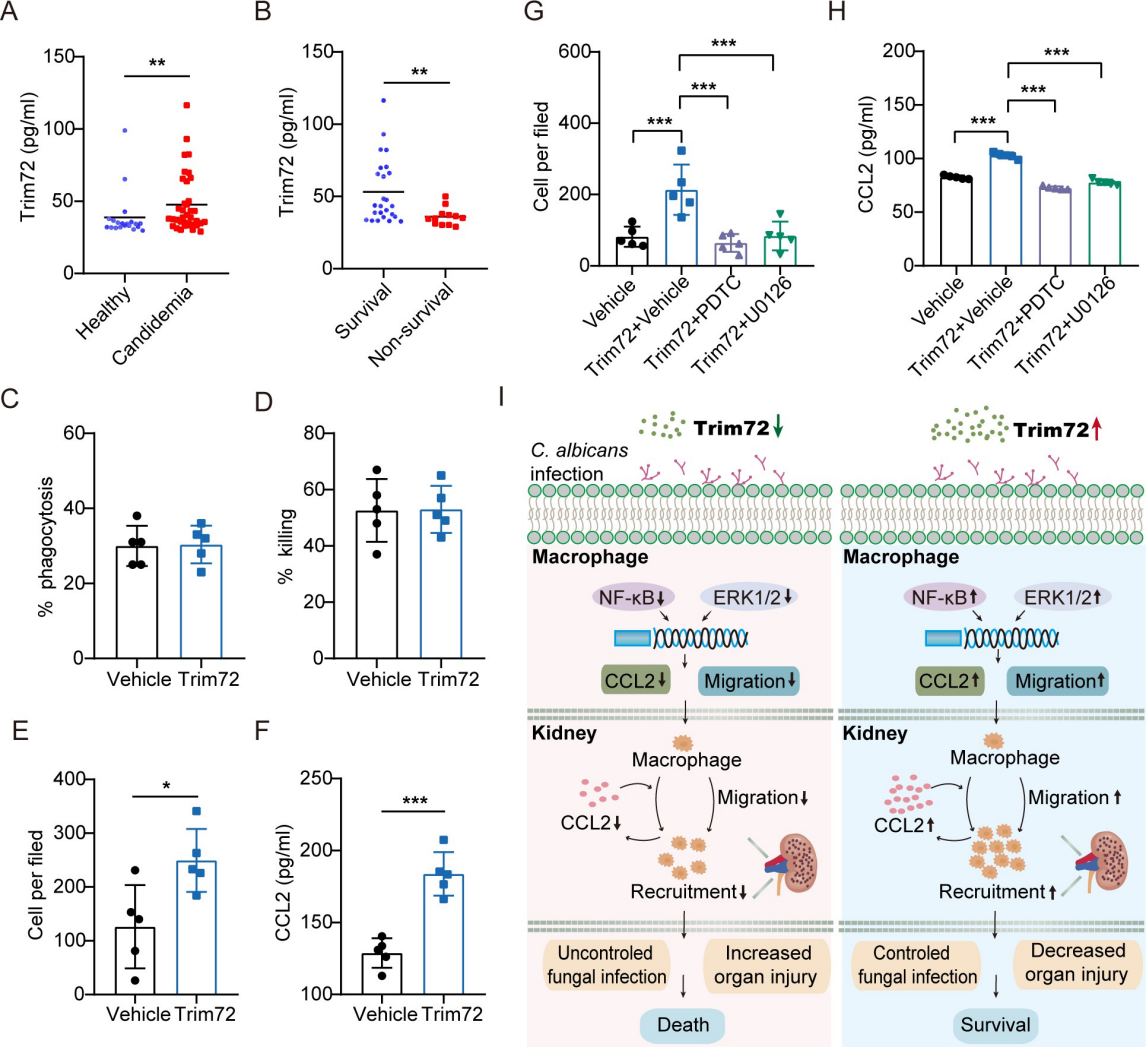
Trim72 augments migration capacity and CCL2 production through NF-κB and ERK1/2 signaling in human macrophages. (A) Trim72 levels were measured by ELISA in serum of patients with candidemia (n = 37) and healthy subjects (n = 20). (B) Trim72 levels in serum of candidemia survivors (n = 25) and candidemia non-survivors (n = 12). (C and D) Rates of phagocytosis (C) and killing (D) for live *C*. *albicans* (MOI = 1) by human monocyte-derived macrophages (hMDMs) treated with vehicle or rmTrim72 (1μg/ml) overnight (n = 5 per group). (E) Migration of hMDMs treated with vehicle or rmTrim72 (1μg/ml) in a transwell migration assay (n = 5 per group). (F) CCL2 levels detected by ELISA in hMDMs treated with vehicle or rmTrim72 (1μg/ml) overnight (n = 5 per group). (G) hMDMs were stimulated with NF-κB inhibitor PDTC (25uM) and ERK1/2 inhibitor U0126 (20uM) or vehicle, followed by rmTrim72 (1μg/ml) or vehicle treatment. Migration of hMDMs in a transwell migration assay (n = 5 per group). (H) CCL2 levels of macrophages with the indicated treatments detected by ELISA (n = 5 per group). (I) Schematic representation of the potential mechanism by which Trim72 protects against *C*. *albicans* infection. Except for clinical research (A, B), data are representative of triplicate independent experiments. Statistical significance was calculated by nonparametric Mann Whitney U test (A, B), two-tailed unpaired t-test (C-F), or one-way ANOVA followed by Dunnett’s multiple comparison test (I, H). Data are presented as mean (A, B) or mean ± SD (C-H). *p < 0.05, **p < 0.01, *** *p* < 0.001.

To test the relevance of our findings in mice to humans, we collected serum to test Trim72 concentrations from 37 patients with candidemia and 20 healthy volunteers. The demographic and clinical characteristics of all subjects were listed in S1 Table. On day of first blood culture positivity for *C*. *albicans*, serum Trim72 concentrations were significantly elevated in candidemia patients versus healthy controls (Fig 10A), and survivors of candidemia patients had significantly higher serum Trim72 levels than non-survivors (Fig 10B).

Next, we investigated whether Trim72 could affect the antifungal activity of human macrophages by isolating human monocytes and inducing them into macrophages. Compared to the vehicle treatment, Trim72 treatment had no effect on fungal phagocytosis and killing in human macrophages (Fig 10C and 10D), whereas Trim72 enhanced cell migration (Fig 10E), and CCL2 production (Fig 10F). In addition, treatment with PDTC or U0126 to inhibit NF-κB or ERK1/2 signaling in human macrophages respectively could impair Trim72-induced cell migration (Fig 10G), and CCL2 production (Fig 10H). Collectively, our results suggest that Trim72 protects against invasive *C*. *albicans* infection by facilitating macrophage recruitment via enhancing cell migration and CCL2 production, which may dependent on NF-κB and ERK1/2 signaling pathways in macrophages (Fig 10I).

## Discussion

Our study identified the critical role of Trim72 in lethal *C*. *albicans* infection and demonstrated its importance in regulating host antifungal immunity. We found that Trim72 concentration was elevated during invasive *C*. *albicans* infection. *Trim72*^*-/-*^ mice were more susceptible to systemic *C*. *albicans* infection, and Trim72 restoration therapy protected mice from systemic *C*. *albicans* infection. Mechanistically, Trim72 facilitated macrophage infiltration and chemokine CCL2 production, which partially mediate Trim72-elicited protection against *C*. *albicans* infection. In vitro, Trim72 boosted cell migration capacity and CCL2 production via NF-κB and ERK1/2 signaling in monocytes/macrophages. Inhibiting NF-κB and ERK1/2 signaling pathways attenuated Trim72-mediated protection against invasive *C*. *albicans* infection. Therefore, our study reveals a previously unrecognized function of Trim72 in antifungal immunity, which protects against *C*. *albicans* infection by regulating macrophage infiltration.

Trim72 has been reported as a potential therapeutic target in a variety of human diseases, such as type 2 diabetes mellitus [36], ischemic heart disease [37], drug-induced liver injury [22] and severe burn injury [38]. Here, we identified Trim72 as a potentially new target for antifungal therapy. The Trim72-deficient mice exhibited increased mortality, organ fungal burden and kidney injury, by reducing macrophage recruitment at 2 days after *C*. *albicans* infection. Exogenous Trim72 supplementation protected mice against lethal *C*. *albicans* infection, decreasing mortality, organ fungal burden and kidney injury. Previous studies have also pointed the important role of Trim72 in some infectious diseases [39,40]. While one study has reported Trim72 as a detrimental factor in pulmonary bacterial infections [26], a discrepancy for that is likely due to the different disease models constructed using different pathogens [41,42].

Macrophages are the first line of defense in antifungal innate immunity by eliminating fungal infections [12,30,43]. Depleting macrophages by clodronate liposome emphasizes the critical role of macrophages in host defense against invasive *C*. *albicans* infection [44]. In our study, Trim72 enhanced monocyte/macrophage recruitment to the infected kidney, and macrophage depletion abrogated Trim72-mediated protection against *C*. *albicans* infection, suggesting that macrophages may be the target cells for Trim72-induced protection against invasive *C*. *albicans* infection. Following fungal infections, monocytes/macrophages are recruited to the site of infection, partially in response to CCL2 [45]. One study highlighted the important role of CCL2-recruited macrophages in fighting microbial infection, by showing that CCL2 neutralization increased pulmonary fungal burden with reduced lung macrophage accumulation during *C*. *neoformans* infection [46]. This mirrors our findings that blocking CCL2 production reversed increased macrophage recruitment induced by Trim72, implying that Trim72 could enhance macrophage recruitment to the infected kidney, partially via increasing CCL2 production. In addition, we found that Trim72 could enhance CCL2 production in macrophages in vitro. Our previous studies have also reported the similar observations, such as BMP9 [47], and progranulin [48], pointing the essential contribution of CCL2-macrophage axis to combat microbial infection. Although macrophages are recognized as a major source of CCL2 [31], other cell types, including fibroblasts, epithelial cells, endothelial cells and vascular smooth muscle cells, may also produce CCL2 [49]. In addition, we found that Trim72 had no effect on *C*. *albicans* phagocytosis and killing in primary macrophages, but could enhance the migratory capacity of macrophages. Trim72 has been reported to promote the migration of other cells, such as human umbilical cord-derived mesenchymal stem cells [50], and corneal epithelia [51], but no study has shown that Trim72 is associated with macrophage migration ability. To our knowledge, our study is the first to report that Trim72 protects against fungal infection by facilitating macrophage recruitment via enhancing cell migration ability and CCL2 production in macrophages. Previous studies have shown that Trim72 plays a multi-organ protective role, mainly due to its tissue repair and regeneration function [52]. Therefore, we speculate that Trim72-mediated tissue repair may also contribute to protection against lethal *C*. *albicans* infection, which requires to be further investigated.

After fungal infections, macrophages recognize the microbes and activate the antifungal signaling pathways, such as the NF-κB and ERK1/2 pathways, which trigger the expression of immunomodulators to perform a range of antifungal killing activities [53,54]. Here our data showed that Trim72 modulated the activation of NF-κB and ERK1/2 pathways in macrophages in response to *C*. *albicans* infection, and that activation of NF-κB and ERK1/2 pathways may correlate with increased cell migration capacity, CCL2 production, and enhanced macrophage recruitment into the infected kidney. Consistent with our results, previous studies have shown that the NF-κB and ERK1/2 pathways can be regulated by a variety of proteins to promote CCL2 secretion by macrophages and macrophage recruitment, such as IL-34 [55], BRD4 [56], and Nesfatin-1 [57]. Other studies have reported that the NF-κB signaling is activated by Trim72 in alveolar macrophages [26], and that Trim72 is essentially involved in cardioprotection by activating the ERK1/2 survival signaling pathways [58,59].

In the development of antifungal therapies for patients with candidemia, it is imperative that these findings from animal studies should be translated into humans. Our study further showed that serum Trim72 were significantly elevated in candidemia patients compared to healthy controls on day of first blood culture positivity for *C*. *albicans*, and that survivors of candidemia patients had significantly higher serum Trim72 levels than non-survivors. In addition, treatment with recombinant human Trim72 had no effects on *C*. *albicans* phagocytosis and killing in human macrophages. However, Trim72 could increase cell migration capacity and augment CCL2 production in human macrophages, which was modulated by NF-κB and ERK1/2 signaling pathways. Therefore, the effects of Trim72 on mouse macrophages can be partially extended to human macrophages, supporting a clinical and translational role of Trim72 in treating humans with fungal infection.

Our study has several limitations. First, the source of Trim72 and the factors regulating Trim72 secretion during *Candida* infection is unclear. Numerous studies have demonstrated that Trim72 can be detected in striated muscle, alveolar epithelial cells and renal proximal tubular epithelium cells and secreted into the blood circulation [16,23], suggesting that these cells may be the potential cellular origin of Trim72 during *Candida* infection. Second, the specific way in which Trim72 acts on macrophages is unclear. Previous studies have shown that Trim72 has been reported to exert its function through various receptors, such as peroxisome proliferation-activated receptor alpha [60], insulin receptor substrate-1 [61], and complement receptor [26]. Based on these findings, we speculate that Trim72 may also act on macrophage surface receptors to induce cell chemotaxis and CCL2 secretion, which requires further investigation. Finally, the number of patients with candidemia in this study was relatively small, so larger multi-centre clinical trials are needed to further evaluate the prognostic value of Trim72.

In summary, we demonstrated the protective role of Trim72 in antifungal responses by recruiting macrophages via enhancing cell migration capacity and upregulating CCL2 producing through NF-kB and ERK1/2 signaling during *Candida* infection. These data expand our understanding of host innate antifungal defense mechanisms and may have broad translational implications for the development of novel antifungal infection approaches.

## Materials and methods

### Ethics statement

All experiments were approved by the Animal Care and Use Committee of the Chongqing Medical University and the Clinical Research Ethics Committee of The First Affiliated Hospital of Chongqing Medical University (Registration number: LLZM-2012-0016, 2021–187 and 2021–655).

### Human study

This study included 37 patients with candidemia aged ⩾18 years who were admitted to The First Affiliated Hospital of Chongqing Medical University from September 2022 to April 2024. The diagnosis of candidemia patients was defined by a positive *C*. *albicans* blood culture based on the 2024 consensus definitions of candidiasis [62]. Demographic and clinical characteristics of all patients were recorded during hospitalization, including age, gender, days of hospitalization, site of infection, common clinical investigations and survival within 30 days of diagnosis of candidemia. Patients with immunosuppressive therapy, pregnancy or breast feeding, malignancy, or viral infections were excluded in this study. Peripheral blood was collected from the patients on the day of the first positive *C*. *albicans* blood culture prior to antifungal therapy, and serum was then separated and stored at −80°C. In addition, 20 age- and sex-matched healthy volunteers were recruited in this study from the Physical Examination Center of The First Affiliated Hospital of Chongqing Medical University as a healthy control group. Informed consent was not required as the study was non-interventional.

### Mice

C57BL/6J *Trim72* knockout (*Trim72*^*-/-*^) mice were purchased from the shanghai model organisms (Shanghai, China) and mouse genotypes were determined by PCR using genomic tail DNA. Primers are listed: P1: TTTCTGGAAGCCTGCTGTGT; P2: TCTGCCCTTTGCCGTGTTAT; P3: TTGCAGTCTGGTGGTGATCT; P4: ATCACCCCGAACCCTTTCTG. Wildtype C57BL/6J mice were purchased from Vital River Laboratory Animal Technology Company (Beijing, China). All mice were housed in the specific pathogen free animal facility. Sex- and age-matched mice were used in all experiments.

### Systemic *C*. *albicans* infection

For in vivo *C*. *albicans* infection, male or female C57BL/6J mice [(8 to 12 weeks old, weighing 20 to 22 g, male or female (1:1)] were intravenously infected with *C*. *albicans* (SC5314) and monitored daily for survival, weight, and disease score (0, bright, alert, responsive; 1, slightly lethargic; 2, lethargic and hunched; 3, very lethargic and shaky; 4, dead) [63]. Fungal load was assessed by plating a series of diluted homogenized organ solutions onto the YPD plates. After 24 hours of incubation at 37°C, CFUs were counted and determined as CFU/g tissue.

### Treatment of mice with recombinant Trim72, and inhibitors of CCL2, NF-κB and ERK1/2 pathways

For in vivo experiments, mouse recombinant Trim72 (catalog OPCA220989, Aviva Systems Biology) was injected intraperitoneally at a dose of 62.5–250 μg/kg 30 minutes prior to *C*. *albicans* infection, and with 31, 62.5, and 125 μg/kg rmTrim72 respectively at 2 days after infection. The CCL2 inhibitor Bindarit (catalogue S3032, Selleck), the NF-κB pathway inhibitor PDTC (catalogue S3633, Selleck) and the ERK1/2 pathway inhibitor U0126 (catalogue S1102, Selleck) were administered intraperitoneally daily at a dose of 100mg/kg, 100mg/kg and 30mg/kg, respectively. Double-distilled water or DMSO was delivered in a similar fashion as a vehicle control.

For in vitro experiments, cells were treated for 2 h with PDTC (25uM) or U0126 (20uM) followed by treatment with mouse recombinant Trim72 (1μg/ml) for the indicated times. Double-distilled water or DMSO was delivered in a similar fashion as a vehicle control.

### Histopathology

For histopathology analysis, kidneys were fixed in 4% buffered formalin. After paraffin embedding, sectioning, renal tissues were stained with hematoxylin and eosin (H&E) (catalog G1120, Solarbio), Periodic-Acid-Schiff (PAS) (catalog BA4080B, Baso), and sliver stain (catalog BA4094, Baso). Stained slides were scanned by a Pannoramic DESK scanner from 3D-HISTECH (Hungary).

### Measurement of renal injury markers

Serum levels of serum creatinine and blood urea nitrogen were assayed by spectrophotometric analysis (modular DP, Roche) according to the protocols of the International Federation of Clinical Chemistry.

### Flow cytometry

Mice were euthanized at the indicated times and organs were removed. After collagenase 4 (Sigma) / DNAse I (Beyotime) digestion of the organs, Fc blocking, samples were stained with antibodies against CD11b (clone M1/70, BD Biosciences), F4/80 (clone T45-2342, BD Biosciences), Ly-6G (clone 1A8, BD Biosciences), Ly-6C (clone AL-21, BD Biosciences), CD3 (clone 500A2, BD Biosciences), NK1.1 (clone PK136, BD Biosciences), CD11c (clone HL3, BD Biosciences), MHC-II (clone M5/114.15.2, BD Biosciences). Then the immune cells were assessed by CytoFLEX (Beckman Coulter) flow cytometry, and analyzed by CytExpert software.

### Immunofluorescence

For immunofluorescence, the kidneys were formalin-fixed, paraffin-embedded and sectioned. After epitope retrieval, blocking endogenous peroxidase and biotin blocking, all slides were blocked with 5% goat serum, prior to overnight incubation with F4/80 primary antibodies at 4°C. After repeated washing, tissue sections were incubated with fluorochrome-conjugated secondary antibodies for 1 hour at room temperature, washed several times and mounted on slides. Images were captured using 3D-HISTECH fluorescence microscopy (Hungary).

### Enzyme-linked immunosorbent assay (ELISA)

Human and mouse Trim72 levels were quantified by human Trim72 (catalog CSB-EL024511HU, Cusabio Biotech) and mouse Trim72 ELISA kits (catalog OKEH01716, Aviva Systems Biology), respectively. Concentrations of IL-1β (catalog 432604), IL-6 (catalog 431304), TNF-α (catalog 430904), IL-4 (catalog 431104), CXCL1 (catalog 447504), and CCL2 (catalog 432704) were determined using the appropriate commercial ELISA kits purchased from Biolegend according to the manufacturer’s instructions.

### In vivo depletion of macrophages

For macrophage depletion, mice were intravenously injected with 200 μl of 5 mg/ml clodronate-containing liposomes (CLD) or empty liposomes (LIPOSOMA) as control 24 hours prior to *C*. *albicans* infection, and reinjected 30 minutes before *C*. *albicans* infection.

### Western blot

Proteins were extracted using RIPA lysis buffer (Beyotime) supplemented with a protease and phosphatase inhibitor cocktail (Thermo Fisher Scientific). Equal amounts of proteins were resolved by sodium dodecyl sulfate (SDS)-polyacrylamide gel electrophoresis (PAGE) followed by transfer to a polyvinylidene fluoride (PVDF) membrane (Millipore). The membrane was blocked with 5% milk for 1h at room temperature, and incubated with Trim72 antibody (catalog YN5445; ImmunoWay), phospho-ERK1/2^Thr202/Tyr204^ antibody (catalog 4370; Cell Signaling Technology), ERK1/2 antibody (catalog 4348; Cell Signaling Technology), phospho-JNK^Thr183/Tyr185^ antibody (catalog 4668; Cell Signaling Technology), JNK antibody (catalog 9252; Cell Signaling Technology), phospho-p38 MAPKThr^180/Tyr182^ antibody (catalog 4511; Cell Signaling Technology), p38 MAPK antibody (catalog 8690; Cell Signaling Technology), phospho-NF-κB p65^Ser536^ antibody (catalog 3033; Cell Signaling Technology), NF-κB p65 antibody (catalog 8242; Cell Signaling Technology), and β-actin (catalog 4967, Cell Signaling Technology) overnight at 4°C. After washing, the membrane was incubated with horseradish peroxidase-conjugated secondary antibodies (catalog ZB-2301, ZSGB-BIO, Beijing, China) at room temperature for 1 h. Protein blots were visualized using an ECL chemiluminescent detection system.

### Quantitative Real-time PCR (qRT-PCR)

Total RNA was isolated using TRIZOL (Invitrogen) and reverse transcribed into cDNA using Prime Script RT reagent kits (Takara). Quantitative PCR was performed using sybr green reagent (MCE). The amounts of transcript were normalized to β-actin. Primers used were listed in S2 Table.

### Isolation of mice primary macrophages

Primary peritoneal macrophages were collected via peritoneal lavage with cold PBS 5 days after intraperitoneal injection of 1mL 4% thioglycolate (catalog LA4590, Solarbio). The cells were counted and cultured in DMEM for 2h, after which non-adherent cells were removed.

For preparation of mice bone marrow–derived macrophages (BMDM), bone marrow was collected from femurs and tibias by flushing with RPMI-1640 media. Bone marrow cells were cultured at RPMI-1640 media with 10% FBS and stimulated with 50 ng/ml Macrophage colony-stimulating factor (M-CSF, catalog 315–02, PeproTech) for 7 days to differentiate into BMDM.

### Culture of human monocyte-derived macrophages (hMDMs)

Human monocytes were isolated from peripheral blood and differentiated into hMDMs with 50 ng/ml M-CSF (catalog 300–25, PeproTech) in 1640 medium. Cells were further used for the indicated assays.

### Phagocytosis and killing assays

For phagocytosis assay of live *C*. *albicans*, cells (5×10^5^) were co-cultured with *C*. *albicans* (MOI = 1) (at t = 0 h) at 37°C for 1h (at t = 2 h), and then cells were washed with PBS to remove unengulfed fungus followed by lysed with double-distilled water. The phagocytosed fungi were quantified by lysate culture. phagocytosis rate was calculated as follows: [number of phagocytosed fungi at t = 2 h] / [number of phagocytosed fungi at t = 0 h] × 100%.

For killing assay, after 2 hours of cocultured of cells with fungi, the unengulfed fungus were washed off using PBS, and then the cells are cultured at 37°C for an additional 2 hours (at t = 4 h) before lysing with double-distilled water. The phagocytosed fungi were quantified by lysate culture. Killing rate was calculated as follows: 100% − [number of phagocytosed fungi at t = 4 h] / [number of phagocytosed fungi at t = 2 h] × 100%.

For phagocytosis assay of HKCA, cells (5×10^5^) were cocultured with FITC (Sigma-Aldrich)-labeled HKCAs (MOI = 1) at 37°C for 2 hours. Adherent fungal cells were quenched with trypan blue (Sigma Aldrich). After cells stained with DAPI for 5min, images were captured using fluorescence microscopy.

### Macrophage migration assay

Primary macrophages (2×10^5^) were plated on the membranes of 6.5mm transwell inserts with 5μm pores (Costar, Corning, NY) using FBS-free media. 10% FBS was added to media in the lower chamber as a chemoattractant. After incubation for 48 h at 37°C, non-migrated cells were removed with cotton swabs. After fixed with 4% paraformaldehyde, stained with 1% crystal violet solution and washed with double-distilled water, the migrated cells were photographed using light microscopy. For each well, four random fields were determined.

### Macrophage scratch assay

Primary macrophages (3×10^6^) were seeded in six-pore plates. A scratch was made in the confluent layer of cells by a sterile 200 μl pipette tip. After three washes, the migration of the cells into the wound area were imaged using light microscopy at indicated time.

### Statistical analysis

Statistical analyses were generated using GraphPad Prism software 8.0.1. All quantitative variables were tested for normality and compared using unpaired two-tailed Student’s t-test, nonparametric Mann Whitney U test or ANOVA analysis. Differences in survival were analyzed by log-rank test. All tests were two-tailed. Data are presented as mean or mean ± SD. P values < 0.05 were accepted as statistically significant.

## Supporting information

S1 Fig Construction of the Trim72 knockout mouse. (A) Schematic of the Trim72 knockout mouse using the clustered regularly interspaced short palindromic repeats (CRISPR) method. (B) Mice were genotyped by PCR using genomic tail DNA. The PCR products were analyzed by agarose gel electrophoresis. Representative images were shown. (C) Representative Western blot analysis images of Trim72 protein expression in kidneys from WT or *Trim72*^*-/-*^ mice. Data are representative of triplicate independent experiments.
(TIF)

S2 Fig Trim72 treatment reduces the fungal load of *C*. *albicans* in organs. *C*. *albicans* fungal load in organs at indicated times after infection (n = 5 per group). Data are representative of triplicate independent experiments. Statistical significance was calculated by two-tailed unpaired t-test or nonparametric Mann Whitney U test. Data are presented as mean ± SD. *p < 0.05.
(TIF)

S3 Fig Trim72 deficiency does not affect immune cell development. (A) Flow cytometry gating strategy determining the indicated innate immune cell lineages. (B) Frequency of indicated innate immune cell lineages and total immune cell counts of bone marrow or spleen of unchanged WT and *Trim72*^*-/-*^ mice (n = 5 per group). Data are representative of triplicate independent experiments. Statistical significance was calculated by two-tailed unpaired t-test. Data are presented as mean ± SD.
(TIF)

S4 Fig The effect of Trim72 deficiency on the host immune response to *C*. *albicans* infection. (A) Flow cytometry gating strategy determining the percentage of CD11b^+^F4/80^+^ macrophages and CD11b^+^Ly6G^+^ neutrophils in the kidneys from WT and *Trim72*^*-/-*^ mice at 2 days after *C*. *albicans* infection. Representative FACS plots from there independent experiments were shown. (B) Representative immunofluorescence pictures of F4/80^+^ macrophages in the kidneys of WT or *Trim72*^*-/-*^ mice at 2 days after *C*. *albicans* infection. Scale bar = 50μm. (C) Cytokine levels in renal tissue homogenates from WT or *Trim72*^*-/*-^ mice detected by ELISA at 2 days after *C*. *albicans* infection (n = 5 per group). Data are representative of triplicate independent experiments. Statistical significance was calculated by two-tailed unpaired t-test. Data are presented as mean ± SD.
(TIF)

S5 Fig The effect of Trim72 treatment on the host immune response to *C*. *albicans* infection. (A) Flow cytometry gating strategy determining the percentage of CD11b^+^F4/80^+^ macrophages and CD11b^+^Ly6G^+^ neutrophils in the kidneys from vehicle-treated and Trim72-treated mice at 2 and 4 days after *C*. *albicans* infection. Representative FACS plots from there independent experiments were shown. (B) Representative immunofluorescence pictures of F4/80^+^ macrophages in the kidneys of vehicle-treated or Trim72-treated mice at 4 days after infection. Scale bar = 50μm. (C) Cytokine levels in renal tissue homogenates from vehicle-treated or Trim72-treated mice detected by ELISA at 2 and 4 days after *C*. *albicans* infection (n = 5 per group). Data are representative of triplicate independent experiments. Statistical significance was calculated by two-tailed unpaired t-test. Data are presented as mean ± SD.
(TIF)

S6 Fig Validation of macrophage depletion. Flow cytometry analysis of CD11b^+^F4/80^+^ macrophages in the kidneys of rmTrim72-treated mice in the presence or absence of macrophages depletion at 2 days after *C*. *albicans* infection (n = 5 per group). CLD, clodronate-containing liposomes; Lipo, empty liposomes. Data are representative of triplicate independent experiments. Statistical significance was calculated by two-tailed unpaired t-test. Data are presented as mean ± SD. **p < 0.01.
(TIF)

S7 Fig Trim72 treatment enhances macrophage migration. (A) Migration of primary peritoneal macrophages treated with rmTrim72 (1μg/ml) or vehicle in a scratch assay. Representative images were shown. Scale bar = 1000 um. (B) Migration of bone marrow-derived macrophages (BMDM) treated with rmTrim72 (1μg/ml) or vehicle in a transwell migration assay (n = 5 per group). Representative images were shown. Scale bar = 1000 um. (C) Relative mRNA expression of integrin gene at 12h after *C*. *albicans* infection in primary peritoneal macrophages pretreated with rmTrim72 (1μg/ml) or vehicle overnight (n = 5 per group). Data are representative of triplicate independent experiments. Statistical significance was calculated by two-tailed unpaired t-test (A-C). Data are presented as mean ± SD. *p < 0.05.
(TIF)

S8 Fig Effect of Trim72 on other cytokine levels. The levels of IFN-a, IFN-β and IFN-γ detected by ELISA in primary peritoneal macrophages treated with vehicle or rmTrim72 (1μg/ml) (n = 5 per group). Data are representative of triplicate independent experiments. Statistical significance was calculated by two-tailed unpaired t-test. Data are presented as mean ± SD.
(TIF)

S9 Fig Effect of Trim72 on JNK and P38 signaling activation after *C*. *albicans* infection in macrophages. (A) Primary peritoneal macrophages pretreated with rmTrim72 (1μg/ml) or vehicle were stimulated with *C*. *albicans* for the indicated periods and analyzed by Western blotting for the indicated signaling molecules. Representative images were shown (n = 3 per group). (B) WT or *Trim72*^*-/-*^ macrophage were stimulated with *C*. *albicans* for the indicated period and analyzed by Western blotting for the indicated signaling molecules. Representative images were show (n = 3 per group). Data are representative of triplicate independent experiments. Statistical significance was calculated by two-tailed unpaired t-test (A, B). Data are presented as mean ± SD.
(TIF)

S1 Table Characteristics of patients with candidemia. Note: Categorical variables are expressed as n (%), and continuous variables were expressed as median (interquartile range). WBC, white blood cell; CRP, C-reactive protein; HR, heart rate; RR, respiratory rate.
(DOCX)

S2 Table Primer sequences used for qRT-PCR. (DOCX)

S1 Data Source file. (XLSX)
