# Supplementary figures and images for: Trim72 is a major host factor protecting against lethal Candida albicans infection
Source: PLoS Pathog. 2024 Nov 25;20(11):e1012747. doi: 10.1371/journal.ppat.1012747 (PMC11627414; doi:10.1371/journal.ppat.1012747)

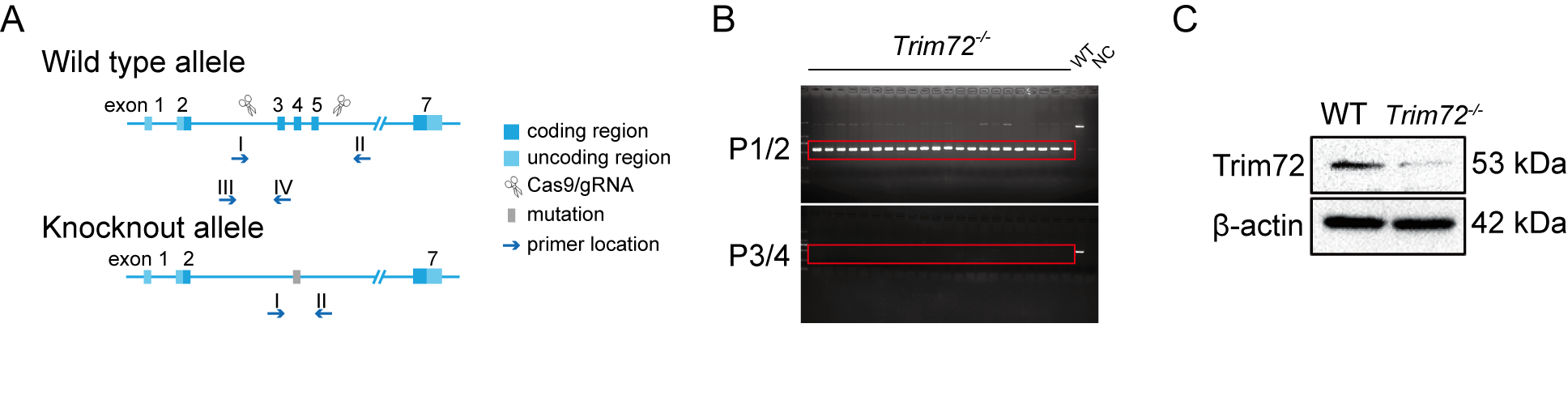

Supplement: S1 Fig — (A) Schematic of the Trim72 knockout mouse using the clustered regularly interspaced short palindromic repeats (CRISPR) method. (B) Mice were genotyped by PCR using genomic tail DNA. The PCR products were analyzed by agarose gel electrophoresis. Representative images were shown. (C) Representative Western blot analysis images of Trim72 protein expression in kidneys from WT or Trim72-/- mice. Data are representative of triplicate independent experiments. (TIF) [file ppat.1012747.s001.tif]

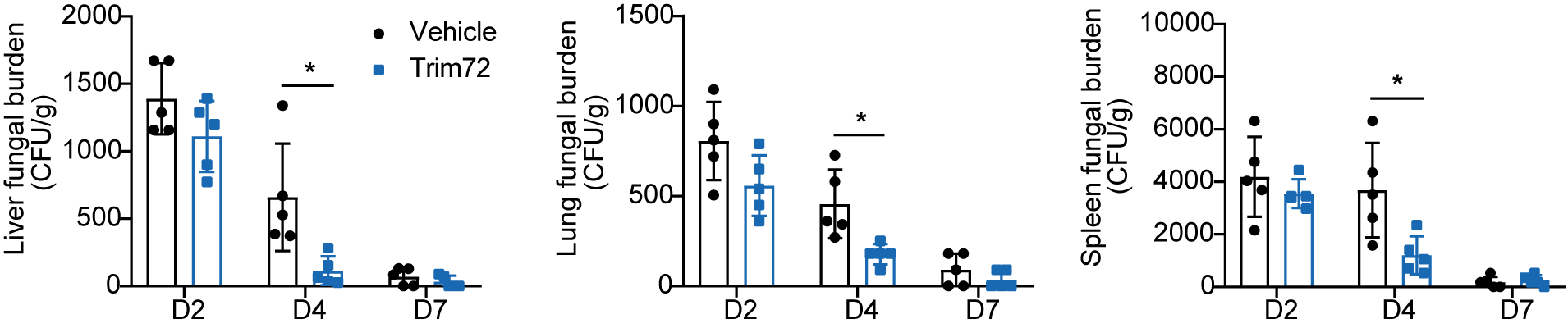

Supplement: S2 Fig — C. albicans fungal load in organs at indicated times after infection (n = 5 per group). Data are representative of triplicate independent experiments. Statistical significance was calculated by two-tailed unpaired t-test or nonparametric Mann Whitney U test. Data are presented as mean ± SD. *p < 0.05. (TIF) [file ppat.1012747.s002.tif]

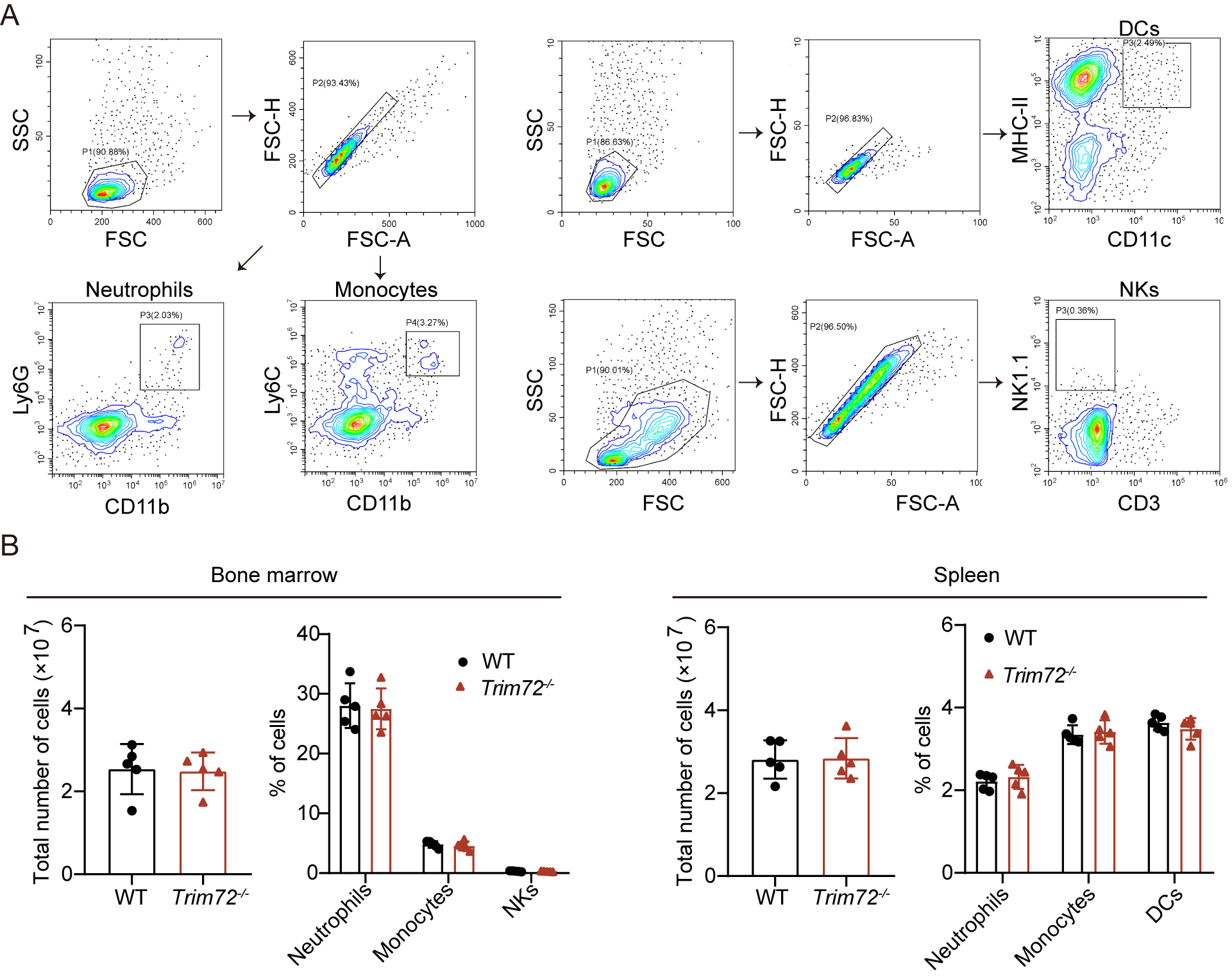

Supplement: S3 Fig — (A) Flow cytometry gating strategy determining the indicated innate immune cell lineages. (B) Frequency of indicated innate immune cell lineages and total immune cell counts of bone marrow or spleen of unchanged WT and Trim72-/- mice (n = 5 per group). Data are representative of triplicate independent experiments. Statistical significance was calculated by two-tailed unpaired t-test. Data are presented as mean ± SD. (TIF) [file ppat.1012747.s003.tif]

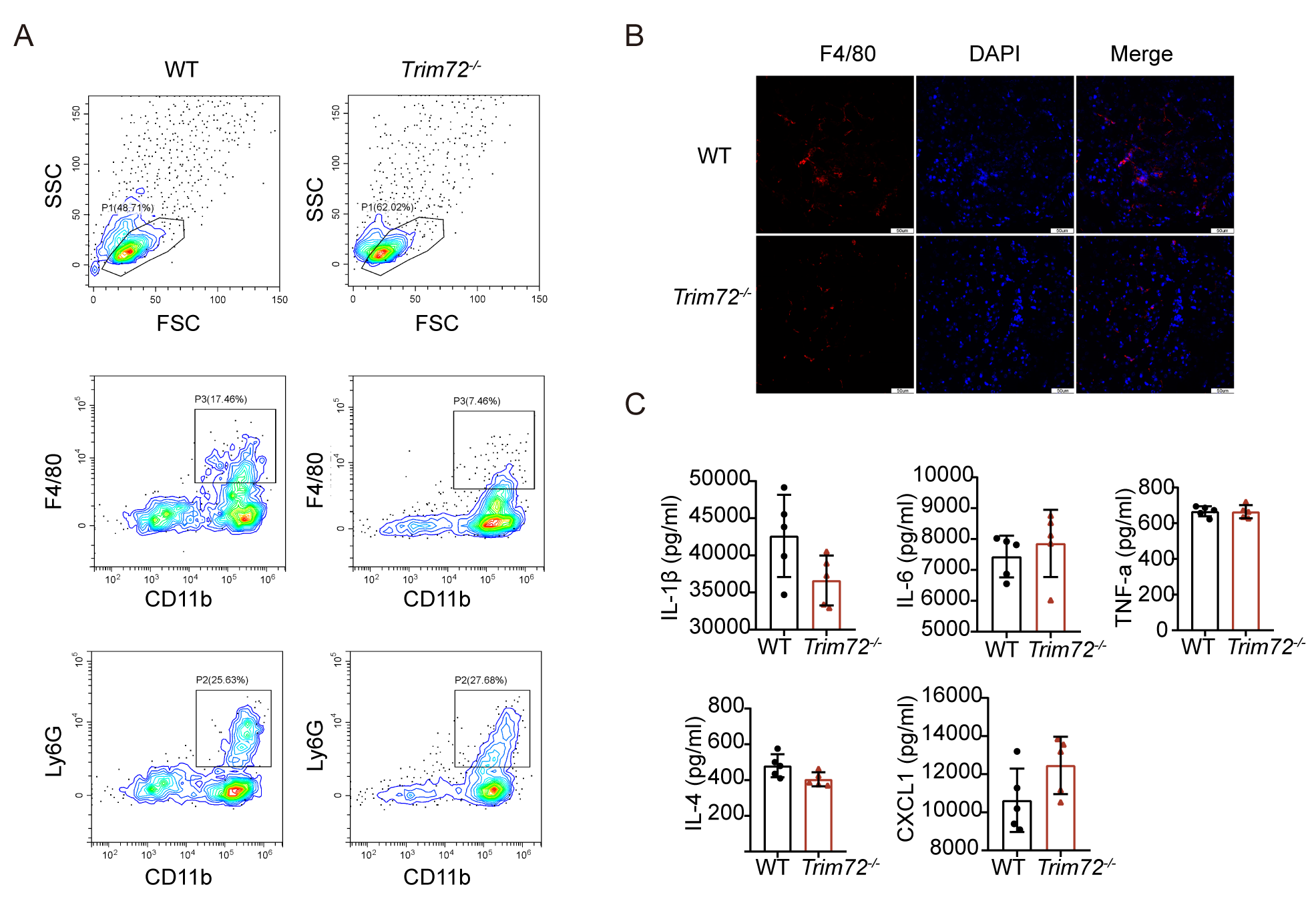

Supplement: S4 Fig — (A) Flow cytometry gating strategy determining the percentage of CD11b+F4/80+ macrophages and CD11b+Ly6G+ neutrophils in the kidneys from WT and Trim72-/- mice at 2 days after C. albicans infection. Representative FACS plots from there independent experiments were shown. (B) Representative immunofluorescence pictures of F4/80+ macrophages in the kidneys of WT or Trim72-/- mice at 2 days after C. albicans infection. Scale bar = 50μm. (C) Cytokine levels in renal tissue homogenates from WT or Trim72-/- mice detected by ELISA at 2 days after C. albicans infection (n = 5 per group). Data are representative of triplicate independent experiments. Statistical significance was calculated by two-tailed unpaired t-test. Data are presented as mean ± SD. (TIF) [file ppat.1012747.s004.tif]

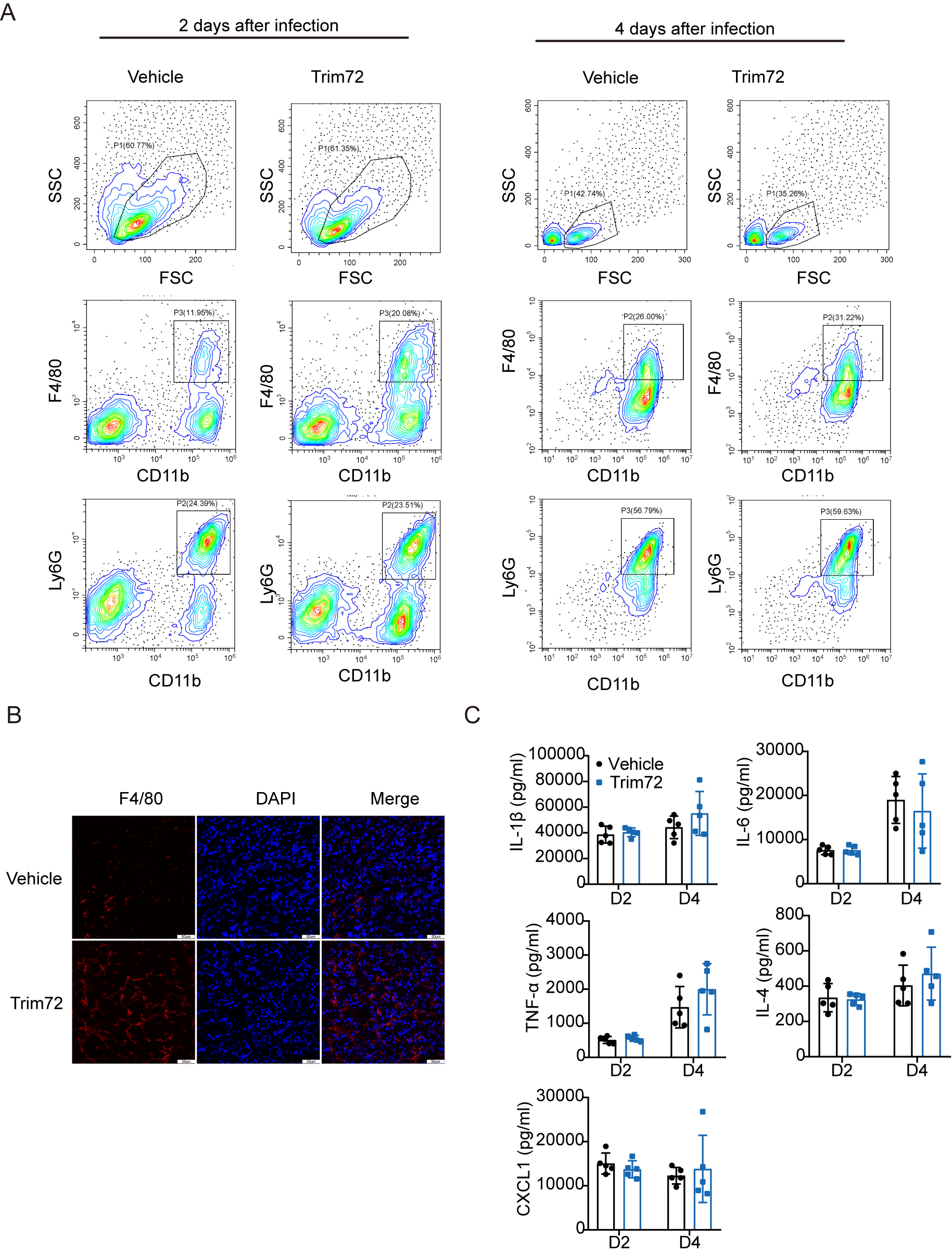

Supplement: S5 Fig — (A) Flow cytometry gating strategy determining the percentage of CD11b+F4/80+ macrophages and CD11b+Ly6G+ neutrophils in the kidneys from vehicle-treated and Trim72-treated mice at 2 and 4 days after C. albicans infection. Representative FACS plots from there independent experiments were shown. (B) Representative immunofluorescence pictures of F4/80+ macrophages in the kidneys of vehicle-treated or Trim72-treated mice at 4 days after infection. Scale bar = 50μm. (C) Cytokine levels in renal tissue homogenates from vehicle-treated or Trim72-treated mice detected by ELISA at 2 and 4 days after C. albicans infection (n = 5 per group). Data are representative of triplicate independent experiments. Statistical significance was calculated by two-tailed unpaired t-test. Data are presented as mean ± SD. (TIF) [file ppat.1012747.s005.tif]

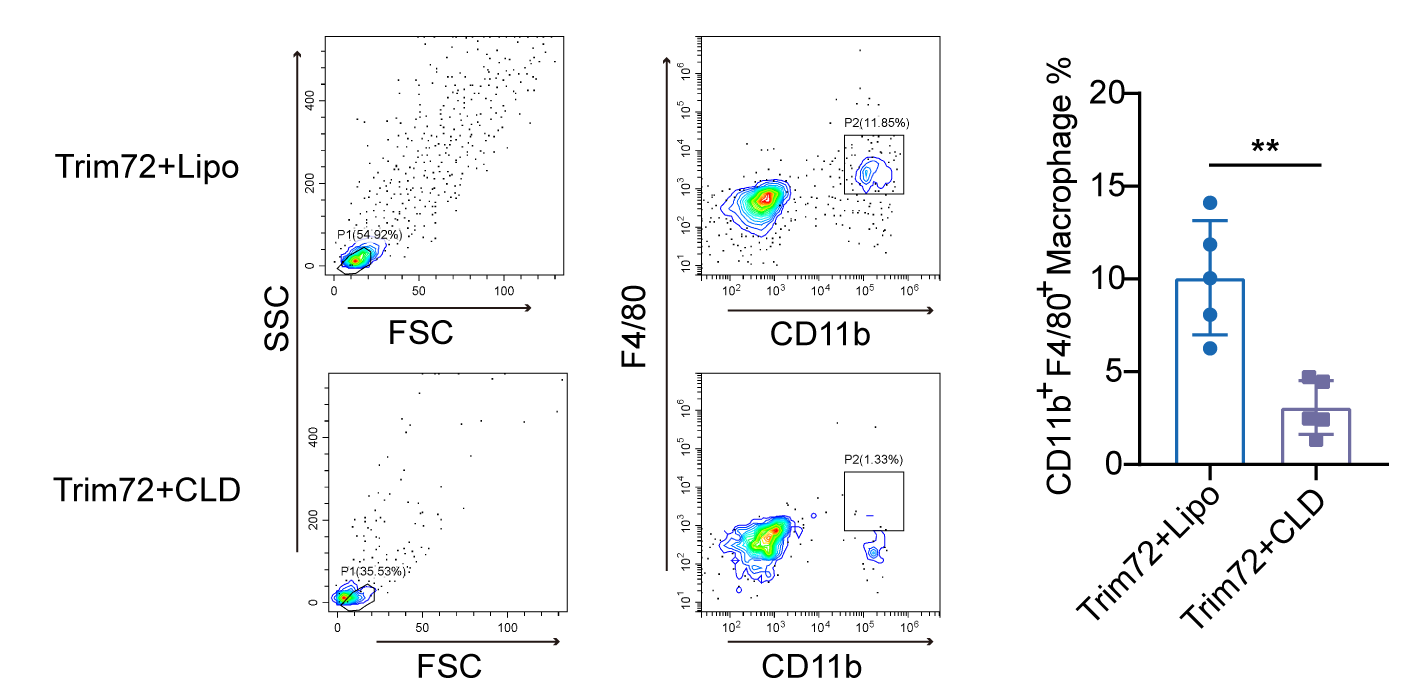

Supplement: S6 Fig — Flow cytometry analysis of CD11b+F4/80+ macrophages in the kidneys of rmTrim72-treated mice in the presence or absence of macrophages depletion at 2 days after C. albicans infection (n = 5 per group). CLD, clodronate-containing liposomes; Lipo, empty liposomes. Data are representative of triplicate independent experiments. Statistical significance was calculated by two-tailed unpaired t-test. Data are presented as mean ± SD. **p < 0.01. (TIF) [file ppat.1012747.s006.tif]

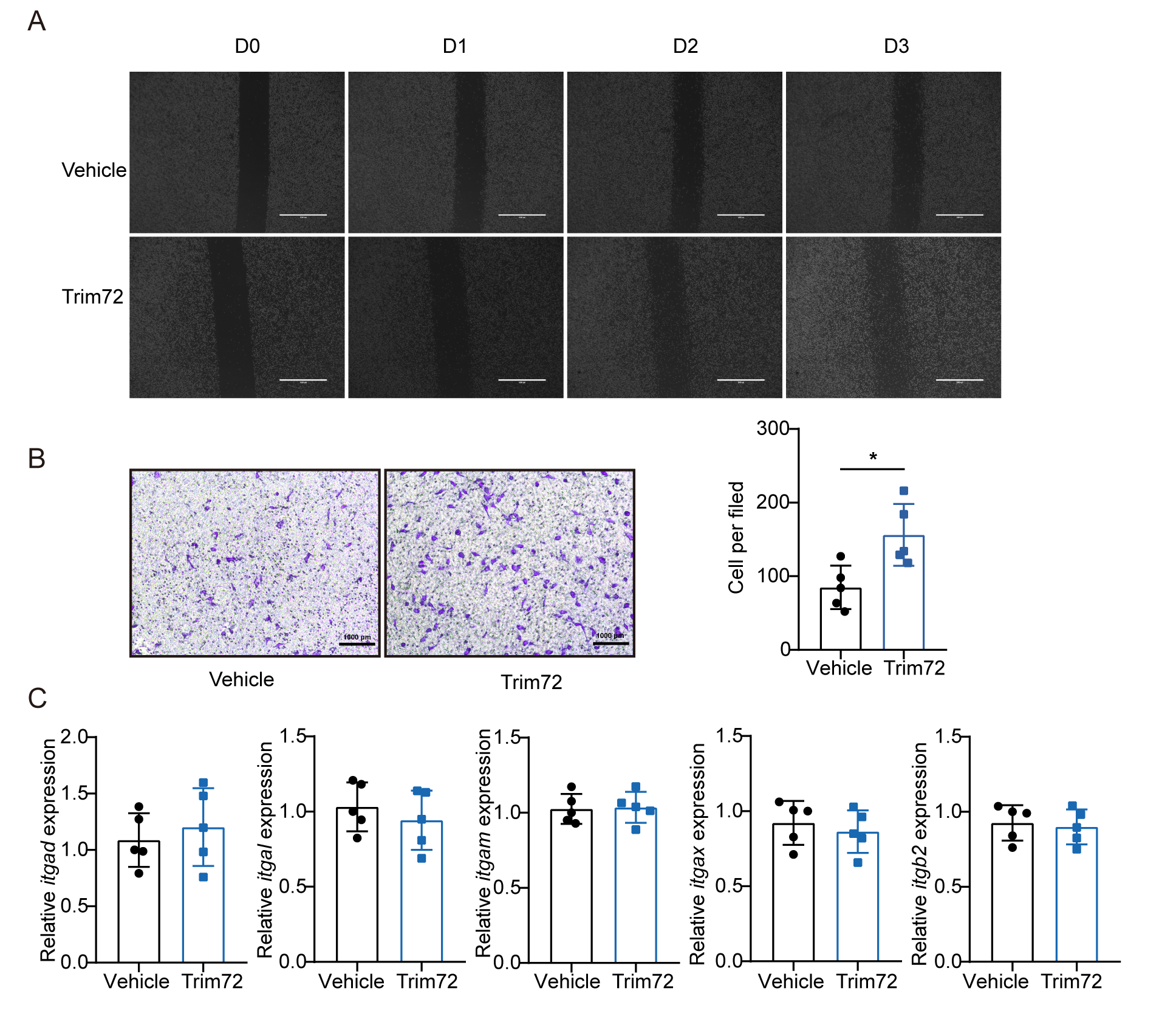

Supplement: S7 Fig — (A) Migration of primary peritoneal macrophages treated with rmTrim72 (1μg/ml) or vehicle in a scratch assay. Representative images were shown. Scale bar = 1000 um. (B) Migration of bone marrow-derived macrophages (BMDM) treated with rmTrim72 (1μg/ml) or vehicle in a transwell migration assay (n = 5 per group). Representative images were shown. Scale bar = 1000 um. (C) Relative mRNA expression of integrin gene at 12h after C. albicans infection in primary peritoneal macrophages pretreated with rmTrim72 (1μg/ml) or vehicle overnight (n = 5 per group). Data are representative of triplicate independent experiments. Statistical significance was calculated by two-tailed unpaired t-test (A-C). Data are presented as mean ± SD. *p < 0.05. (TIF) [file ppat.1012747.s007.tif]

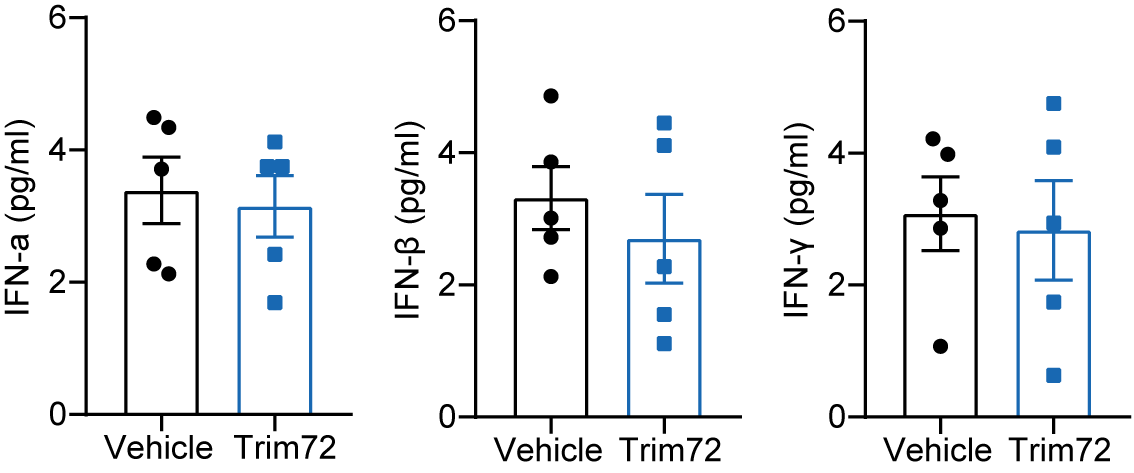

Supplement: S8 Fig — The levels of IFN-a, IFN-β and IFN-γ detected by ELISA in primary peritoneal macrophages treated with vehicle or rmTrim72 (1μg/ml) (n = 5 per group). Data are representative of triplicate independent experiments. Statistical significance was calculated by two-tailed unpaired t-test. Data are presented as mean ± SD. (TIF) [file ppat.1012747.s008.tif]

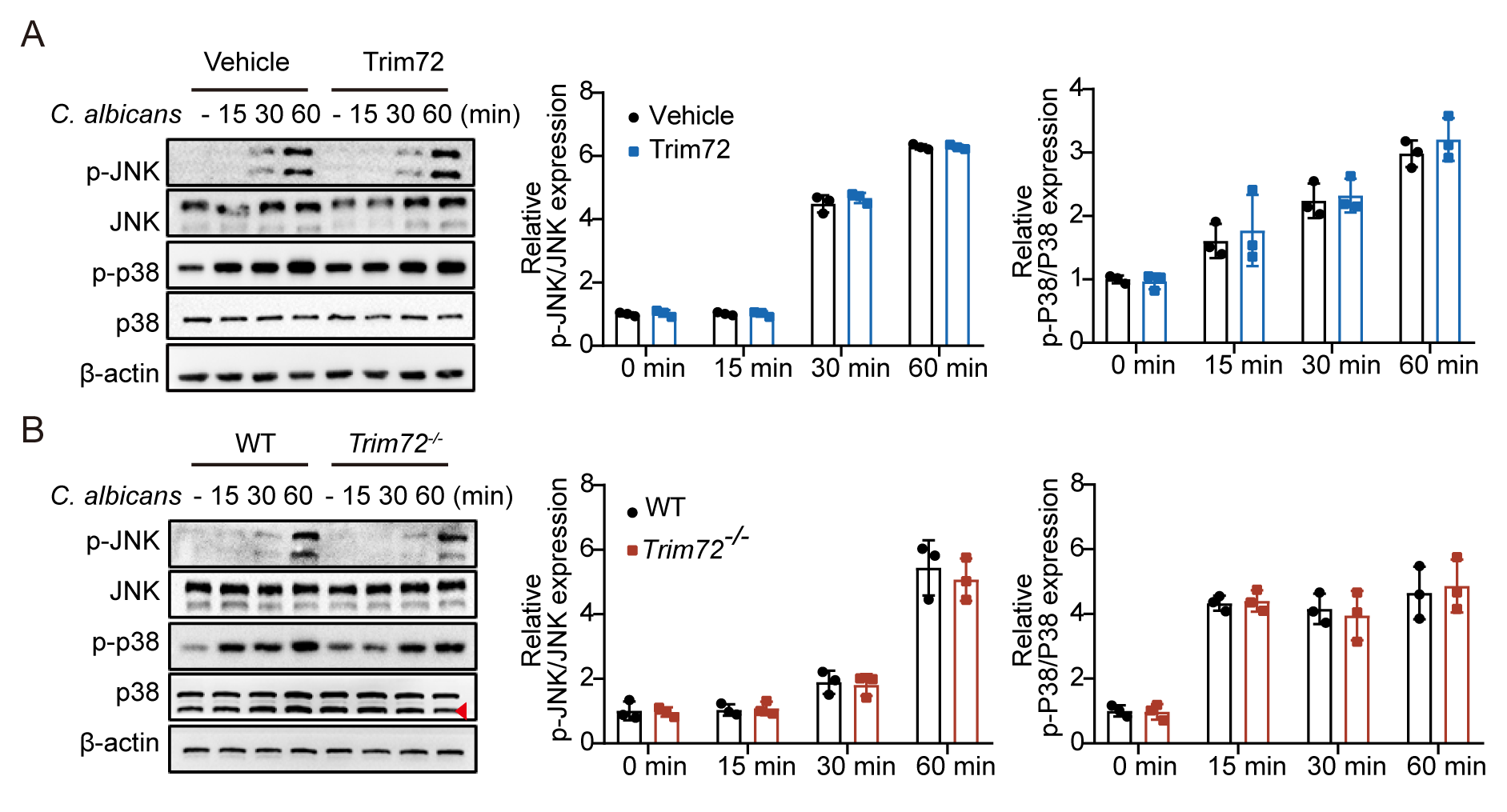

Supplement: S9 Fig — (A) Primary peritoneal macrophages pretreated with rmTrim72 (1μg/ml) or vehicle were stimulated with C. albicans for the indicated periods and analyzed by Western blotting for the indicated signaling molecules. Representative images were shown (n = 3 per group). (B) WT or Trim72-/- macrophage were stimulated with C. albicans for the indicated period and analyzed by Western blotting for the indicated signaling molecules. Representative images were show (n = 3 per group). Data are representative of triplicate independent experiments. Statistical significance was calculated by two-tailed unpaired t-test (A, B). Data are presented as mean ± SD. (TIF) [file ppat.1012747.s009.tif]
