# Supplementary material for: Trim72 is a major host factor protecting against lethal Candida albicans infection
Source: PLoS Pathog. 2024 Nov 25;20(11):e1012747. doi: 10.1371/journal.ppat.1012747 (PMC11627414; doi:10.1371/journal.ppat.1012747)
Supplement: S1 Table — Note: Categorical variables are expressed as n (%), and continuous variables were expressed as median (interquartile range). WBC, white blood cell; CRP, C-reactive protein; HR, heart rate; RR, respiratory rate. (DOCX) [file ppat.1012747.s010.docx]

**S1 Table. Characteristics of patients with candidemia.**

| **Characteristics** | **All patients with candidemia**  **n=37** | **Survivor patients with candidemia**  **n=25** | **Non-survivor patients with candidemia**  **n=12** |
| --- | --- | --- | --- |
| Male sex | 23 (62.16%) | 16(64.00%) | 7(58.33%) |
| Age | 66(55,76) | 61(55,72) | 71.5(63.25, 86.75) |
| Days of hospitalization | 25 (13.5, 44) | 25 (15, 41.50) | 25 (9, 73.25) |
| **Infection site,** |  |  |  |
| Urinary | 4(10.81%) | 3(12%) | 1(8.33%) |
| Respiratory | 11(29.73%) | 7 (28%) | 4(33.33%) |
| Abdominal | 4(10.81%) | 4(16%) | 0(0%) |
| Multi-site | 5(13.51%) | 4(16%) | 1(8.33%) |
| Other | 13(35.14%) | 7(28%) | 6(50%) |
| **Parameters at positive blood culture** | | |  |
| WBC, 109/L | 9.16(4.55, 13.18) | 8.37(3.96, 12.78) | 11.28(7.68, 19.05) |
| CRP, mg/L | 70.95(18.5, 118.8) | 80.30(13.10, 117.0) | 68.30(55.10, 137) |
| Procalcitonin, ng/ml | 0.74(0.45, 1.85) | 0.87(0.45, 1.715) | 0.59(0.45, 1.88) |
| Blood urea nitrogen mmol/L | 8.70(6.75, 19.65) | 8.1 (6.55, 14.9) | 16.55 (7.13, 27.65) |
| Serum creatinine umol/L | 97 (63.5, 141.5) | 91 (63.5, 133) | 105 (63.25, 192.8) |
| Serum bilirubin umol/L | 15.70 (8.55, 38.85) | 15.70 (8.55, 38.85) | 23.10 (8.88, 102.8) |
| Platelet (109/L) | 144 (48, 236) | 168 (71, 241.5) | 112 (34, 204) |

Note: Categorical variables are expressed as n (%), and continuous variables were expressed as median (interquartile range). WBC, white blood cell; CRP, C-reactive protein; HR, heart rate; RR, respiratory rate.
