# Supplementary material for: Trim72 is a major host factor protecting against lethal Candida albicans infection
Source: PLoS Pathog. 2024 Nov 25;20(11):e1012747. doi: 10.1371/journal.ppat.1012747 (PMC11627414; doi:10.1371/journal.ppat.1012747)
Supplement: S2 Table — (DOCX) [file ppat.1012747.s011.docx]

**S2 Table. Primer sequences used for qRT-PCR.**

| **Gene** | **Forward Primer (5’>3’)** | | **Reverse Primer (5’>3’)** |
| --- | --- | --- | --- |
| *Ngal* | TCAAGGACGACAACATCATCTTCT | CTCCAGATGCTCCTTGGTATGG | |
| *Itgb2* | TTTCGGCACGTGCTCAAG | TTGCCGACCTCTGTCTGAAAC | |
| *Itgad* | GGAACCGAATCAAGGTCAAGT | ATCCATTGAGAGAGCTGAGCTG | |
| *ItgaL* | CCCGCTTGGTCGGTTTG | CAGTCAGCCTATCCCCATTGA | |
| *Itgam* | TCCGGTAGCATCAACAACAT | GGTGAAGTGAATCCGGAACT | |
| *Itgax* | CTGGATAGCCTTTCTTCTGCT | GCACACTGTGTCCGAACTCA | |
| *β-actin* | CATTGCTGACAGGATGCAGAAGG | TGCTGGAAGGTGGACAGTGAGG | |
